# Recent Advances in Flexible Sensors for Neural Interfaces: Multimodal Sensing, Signal Integration, and Closed-Loop Feedback

**DOI:** 10.3390/bios15070424

**Published:** 2025-07-02

**Authors:** Siyi Yang, Xiujuan Qiao, Junlong Ma, Zhihao Yang, Xiliang Luo, Zhanhong Du

**Affiliations:** 1 The Brain Cognition and Brain Disease Institute (BCBDI), Shenzhen Institutes of Advanced Technology, Chinese Academy of Sciences, Shenzhen 518055, China; sy.yang2@siat.ac.cn (S.Y.); majlong@mail2.sysu.edu.cn (J.M.); e1143634@u.nus.edu (Z.Y.); 2 Guangdong Provincial Key Laboratory of Brain Connectome and Behavior, Shenzhen Institute of Advanced Technology, Chinese Academy of Sciences, Shenzhen 518055, China; 3 CAS Key Laboratory of Brain Connectome and Manipulation, Shenzhen-Hong Kong Institute of Brain Science, Shenzhen Institute of Advanced Technology, Chinese Academy of Sciences, Shenzhen 518055, China; 4 University of Chinese Academy of Sciences, Beijing 101408, China; 5 Key Laboratory of Optic-Electric Sensing and Analytical Chemistry for Life Science, Ministry of Education, College of Chemistry and Molecular Engineering, Qingdao University of Science and Technology, Qingdao 266042, China; qiaoxiujuan@qust.edu.cn

**Keywords:** flexible sensors, neural interfaces, multimodal sensing, electrochemical detection, closed-loop systems, nanomaterials

## Abstract

The rapid advancement of flexible sensor technology has profoundly transformed neural interface research, enabling multimodal information acquisition, real-time neurochemical and electrophysiological signal monitoring, and adaptive closed-loop regulation. This review systematically summarizes recent developments in flexible materials and microstructural designs optimized for enhanced biocompatibility, mechanical compliance, and sensing performance. We highlight the progress in integrated sensing systems capable of simultaneously capturing electrophysiological, mechanical, and neurochemical signals. The integration of carbon-based nanomaterials, metallic composites, and conductive polymers with innovative structural engineering is analyzed, emphasizing their potential in overcoming traditional rigid interface limitations. Furthermore, strategies for multimodal signal fusion, including electrochemical, optical, and mechanical co-sensing, are discussed in depth. Finally, we explore future perspectives involving the convergence of machine learning, miniaturized power systems, and intelligent responsive materials, aiming at the translation of flexible neural interfaces from laboratory research to practical clinical interventions and therapeutic applications.

## 1. Introduction

Billions of neurons collectively form neural networks, transmitting information via electrical and chemical signals, thus facilitating essential biological activities, such as perception, learning, and memory. Significant advancements have been achieved in the development of brain–computer interface (BCI) technologies, which establish direct communication pathways between neural activities and external systems and are regarded as pivotal tools that could revolutionize human–technology interactions. Furthermore, BCIs hold considerable promise for the treatment of neurological disorders, offering unprecedented opportunities for clinical applications and therapeutic interventions [1].

Neurotransmitters serve as essential chemical messengers responsible for transmitting and balancing specific signals between neurons and other cell types. These molecules play a critical role in physiological processes, such as heart rate, respiration, and sleep, as well as higher cognitive functions, including learning, memory, consciousness, and emotional regulation. Imbalances in neurotransmitter levels can lead to a variety of disorders, encompassing neurodegenerative diseases, psychiatric conditions, such as depression and schizophrenia, and neurological disorders, including epilepsy and stroke [2]. Real-time monitoring of dynamic changes in neurochemicals in the living brain remains essential for elucidating the molecular mechanisms underlying neurophysiological and pathological processes [3]. Conventional bioelectronic devices are typically rigid and non-stretchable, whereas biological tissues and organs exhibit inherent softness and dynamic characteristics. Such mechanical mismatches between bioelectronic devices and biological tissues can cause tissue injury, immune reactions, and chronic inflammation [4].

Rigid materials are susceptible to internal fracture, which can impair the performance and stability of sensors, rendering them inadequate for the evolving requirements of flexible sensing technologies. One of the first flexible invasive electrodes fabricated from compliant materials was reported in 2001 [5]. In recent years, flexible electrodes fabricated from soft materials have gained increasing popularity as alternatives to traditional rigid electrodes due to their superior conformability, higher signal-to-noise ratio (SNR) potential, and broader application scope [6]. Novel structural designs and innovative material approaches, such as thinning bulk rigid materials and transforming them into nanostructures, have significantly enhanced mechanical flexibility [7]. Moreover, patterning thin (<1 μm) inorganic device layers into serpentine geometries enables inherently non-stretchable materials to achieve low bending stiffness and stretchability, thereby enhancing their compatibility with flexible sensing and monitoring applications, as well as requirements for flexible and stretchable electronics [4].

With the iterative advancement of neural interface technologies, multimodal integration has become crucial for overcoming the limitations associated with single-signal detection. Current systems have successfully combined electrophysiological signal acquisition with mechanical sensing, utilizing flexible electrode arrays capable of simultaneously recording neuronal electrical activity and local stress variations. Furthermore, the introduction of microfluidic chips integrated with nano-piezoresistive sensing units enables real-time monitoring of fluid pressure fluctuations induced by neurotransmitter diffusion, providing spatiotemporal complementarity with electrical signals [8]. The interdisciplinary convergence of neuroscience and materials science has expanded the focus of neural interface technologies beyond signal acquisition and stimulation, increasingly emphasizing their contributions to neural repair and functional restoration. The potential of flexible neural interfaces in modulating local immune responses, promoting nerve regeneration, and facilitating functional recovery offers valuable insights for the bioadaptive design of next-generation flexible sensors [9].

Breakthrough advancements in flexible neural interface technology are driving the evolution of brain–computer interaction from unidirectional recording toward closed-loop systems [10]. Machine learning and artificial intelligence (AI) methods are increasingly being applied to patients in intensive care units, enabling dynamic adjustment of electrical stimulation parameters and the development of seizure risk prediction models, thereby significantly improving the timeliness of interventions. When elevated intracranial pressure is detected, AI systems may also recommend treatment strategies, such as the administration of mannitol. With appropriate training, AI systems can further assess the outcomes of treatments (e.g., changes in plasma osmolality following osmotherapy) and propose corrective measures [11]. Mechanical–electrical–chemical synergistic regulation strategies have reduced motor function recovery time by 40% in animal studies. Implantable closed-loop systems have achieved bidirectional interaction in spinal cord injury rehabilitation by adaptively adjusting both electrical stimulation intensity and drug release rates, thereby overcoming the limitations of traditional open-loop mechanical response models [12]. Multimodal integration provides multidimensional information support for closed-loop regulation [13]. Flexible electrode arrays enable simultaneous acquisition of electrophysiological signals and mechanical stress variations. When combined with nano-piezoresistive sensors embedded in microfluidic chips, these systems achieve in situ monitoring of neurotransmitter diffusion dynamics [14]. The integration of optogenetics further empowers cell-type-specific neuromodulation. For example, closed-loop optical stimulation suppresses abnormally firing neurons in the hippocampal region while activating inhibitory interneurons, effectively blocking epileptic seizure cascades in animal models. This electro-optical hybrid closed-loop system reduces response latency to 50 ms and enhances spatiotemporal resolution by two orders of magnitude [15]. Furthermore, advanced flexible closed-loop systems decode motor cortical signals to drive multichannel epidural spinal cord stimulation. By incorporating biomechanical sensors for real-time gait feedback, they enable adaptive reconstruction of motor functions. Experimental validations demonstrate significant performance improvements in neural intervention efficiency and precision compared to conventional open-loop approaches. With advancements in technology [16], intelligent closed-loop interfaces equipped with autonomous learning capabilities are poised to drive transformative changes in therapeutic paradigms, particularly in applications, such as deep brain stimulation for Parkinson’s disease and neuroregulation strategies for depression [17].

In this review, we focus on recent advances in flexible sensors for neural interfaces, with particular emphasis on their critical roles in multimodal information acquisition, real-time neural signal analysis, and closed-loop feedback regulation. We first systematically summarize the latest strategies in flexible materials and microstructural design, and analyze approaches for the co-collection of electrophysiological, mechanical, and chemical signals on a single platform. We then discuss the advantages and challenges of neurotransmitter detection and high spatiotemporal resolution electrochemical sensing technologies for dynamic in vivo monitoring. Recognizing that single-function devices are insufficient to meet the demands of complex neural mechanism studies and clinical applications. Recognizing that single-function devices are insufficient to meet the demands of complex neural mechanism studies and clinical applications [18], we further explore the construction strategies of multimodal integrated systems, including electrochemical–optical synergy, multisignal fusion, and wireless communication modules. Finally, we highlight the frontier applications of closed-loop control systems in neural modulation and envision future development directions integrating machine learning, miniaturized power systems, and intelligent responsive materials, aiming to promote the practical and intelligent advancement of flexible neural interface technologies.

## 2. Recent Advances in Materials and Structural Designs for Flexible Sensors in Neural Interfaces

### 2.1. Flexible Functional Materials

Interfacial materials play a crucial role in determining the performance of flexible neural interfaces. Unlike traditional rigid electrodes, flexible electrodes are typically fabricated from composite materials, consisting of a flexible substrate combined with functional materials. Through specialized structural designs, novel neural interfaces with high sensitivity, high selectivity, and excellent conformability can be developed.

#### 2.1.1. Carbon-Based Materials

Carbon-based materials have important applications in the electrodes of electrochemical sensors, with carbon nanotubes and graphene being the most extensively studied representatives across various fields [19]. In neuroscience, the high electrical conductivity and large specific surface area of carbon-based nanomaterials make them ideal candidates for detecting neuroelectrochemical signals. Moreover, with the growing demand for multimodal sensing, carbon-based nanomaterials exhibit unique advantages in neural interfaces due to their excellent electrical properties and biocompatibility. Li et al. systematically reviewed the applications of carbon nanomaterials in implantable brain–computer interfaces for sensing and stimulation, highlighting the cutting-edge integration of materials science and neural engineering [20].

**Figure 1:**
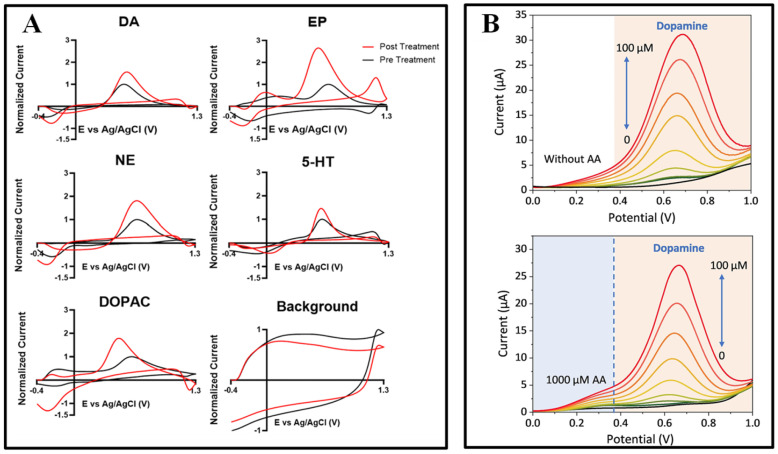
(**A**) Fast-scan cyclic voltammetry (FSCV) results for CFME before and after treatment. Data were collected with CFME before (black) and after (red) electrochemical treatment. (**B**) Square wave voltammetry (SWV) responses for different DA concentrations: without AA and with 1000 μM AA. The potential window for DA is highlighted in orange, while the potential window for AA is highlighted in blue. Reprinted with permission from [22] Copyright © 2024, Analyst.

Ashley E. Ross and colleagues developed graphene fiber microelectrodes (GFMEs) synthesized via a hydrothermal method using graphene oxide (GO) and reduced graphene oxide (rGO). The GO and rGO fiber electrodes demonstrated a significantly higher sensitivity toward dopamine detection (1.54 nA/Μm, in Tris buffer) compared to conventional carbon fiber microelectrodes (CFMEs, 0.41 nA/μM, in Tris buffer). This improvement is attributed to the GFMEs’ faster electron transfer rates (indicated by a smaller ΔEp), higher redox cycling efficiency (Ir/Io approaching 1), and superior antifouling capability against serotonin compared to CFMEs [21]. Although graphene fiber microelectrodes exhibit excellent sensitivity, there remains room for optimization in the dynamic regulation of their surface functional groups. Through chemical modifications that adjust surface charge distribution, their applicability in complex biological environments can be further expanded. Given the presence of various functional groups on carbon-based material surfaces, surface modification can enhance their potential as electrochemical sensing materials by improving antifouling properties, enhancing selective responsiveness, and further boosting electrochemical detection performance. B. Jill Venton and colleagues performed electrochemical treatments using KOH on carbon fiber microelectrodes (CFMEs) and carbon nanotube yarn microelectrodes (CNTYMEs), resulting in approximately a twofold increase in CFME sensitivity and a two- to fourfold enhancement in CNTYME sensitivity toward cationic neurotransmitters. The response to DOPAC, an anionic metabolite, showed minimal improvement due to repulsion by the negatively charged nanogap surfaces. Fast-scan cyclic voltammetry (FSCV) results demonstrated the responses of CFMEs before and after treatment to 1 μM concentrations of dopamine (DA), epinephrine (EP), norepinephrine (NE), serotonin (5-HT), and 20 μM DOPAC (Figure 1A). Compared to a KOH-treated CNTYME, there is significantly more fouling on the untreated electrode Thus, the KOH treatment adds oxide groups, which lead to antifouling properties for serotonin [22].

Although surface engineering of carbon-based materials can significantly enhance their sensing performance, their catalytic activity remains limited in certain complex biological environments. This challenge has driven researchers to explore metal-based materials with superior catalytic properties. Through nanostructure design and composite material strategies, further breakthroughs in sensitivity and selectivity have been achieved.

#### 2.1.2. Metal-Based Materials

In addition to carbon-based materials, metal materials are also important candidates for electrochemical sensing due to their excellent conductivity and catalytic properties. However, because traditional metal electrodes typically exhibit low flexibility and poor toughness, they are generally utilized in the form of nanometal composites for flexible neural electrodes. By loading nanometal particles, nanorods, or depositing nanometal networks onto flexible conductive substrates, flexible neural electrodes can achieve enhanced selective catalytic performance, improved anti-interference capabilities, and, to some extent, increased the electrode surface area, thereby boosting electrochemical detection capabilities.

Jiang, Yan’s team developed a novel electrode for an electrochemical sensor based on gold nanorods (AuNRs) combined with a ZIF-8 (Zeolitic Imidazolate Framework-8) core–shell structure (AuNR@ZIF-8) for the detection of DA and 5-HT. The high surface area of ZIF-8 provides abundant adsorption sites, while the excellent conductivity of AuNRs facilitates electron transfer, synergistically enhancing sensitivity. As a result, AuNR@ZIF-8 achieved detection limits of 0.03 μM for DA and 0.007 μM for ST, with linear ranges of 0.1–50 μM and 0.1–25 μM, respectively. Furthermore, the electrode exhibited high selectivity against interference from K^+^, Na^+^, and ascorbic acid (AA), and its performance remained unaffected by human serum albumin (HSA) adsorption [23].

Although core–shell structures enhance selectivity, challenges, such as nonspecific protein adsorption in real biological samples, still need to be addressed. Combining porous substrates with selective coatings offers an effective strategy to further optimize antifouling performance. Qiuping Wei and colleagues developed an antifouling, high-sensitivity nano-porous diamond sensing interface (NanoDiaSens) capable of specifically detecting dopamine (DA) in human serum while eliminating interference from substances, such as ascorbic acid (AA). By electrodepositing gold nanoparticles (AuNPs, ~44 nm) within the nanoporous boron-doped diamond (pBDD) matrix, catalytic activity was significantly enhanced. A nafion film was subsequently coated onto an electrode surface to repel negatively charged interfering species, further improving selectivity. In complex environments containing human serum and high concentrations of AA (1000 μM), the electrode achieved highly selective dopamine detection with a peak separation (ΔEp) of up to 440 mV (as shown in Figure 1B). Owing to the incorporation of gold nanoparticles, the electrode exhibited a sensitivity of 1.5 μA·μM^−1^·cm^−2^ and a detection limit of 68 nM [22].

The main limitation of solid-state metallic nanostructures lies in their tendency to fracture under mechanical deformation, leading to failure of the conductive network. The emergence of liquid metals offers a new approach to address this issue. Their dynamic flow characteristics not only accommodate complex deformations but also open possibilities for multifunctional integration. In addition to commonly used metals, such as Au, Ag, and Pt, liquid metals have achieved significant progress in flexible electrochemical sensing electrodes due to their unique fluidity and self-healing properties. Compared to solid metals, liquid metals can better adapt to substrate deformations under flexible conditions, thereby mitigating the effects of conductive network damage caused by motion.

In addition, gallium-based liquid metals have good biocompatibility. Foremny et al. [24] systematically evaluated the biocompatibility of Galinstan liquid metal and silicone rubber composites in implant applications. The study confirmed through in vitro cell experiments that the materials treated with steam sterilization showed a cell survival rate of more than 100% in the WST-1 metabolic activity test, indicating that it not only has no cytotoxicity but also may promote cell proliferation. The LDH cytotoxicity test showed that the cell damage rate in the Galinstan-silicone rubber group was less than 30%, which was close to that of the negative control group. In addition, the material was effectively sterilized by steam before implantation to eliminate microbial contamination, and no significant inflammatory response was triggered after implantation. This study proved for the first time that the Galinstan-silicone rubber composite system complies with the ISO 10,993 biocompatibility standard, providing a key safety basis for the clinical application of implantable devices, such as flexible nerve electrodes. Zhao et al. [25] discussed in detail the compatibility characteristics of eutectic gallium-indium alloy (EGaIn) in the biomedical field. In vitro experiments have shown that when EGaIn nanoparticles are co-cultured with 4T1 breast cancer cells and McA-RA7777 liver cancer cells, the relative survival rate of the cells remains above 100%, and they still maintain low toxicity under the action of alternating magnetic fields. The culture experiments of human cells (HeLa cells and adipose-derived stem cells) further verified their biological safety, and no abnormalities were observed in cell morphology and survival rate within 3 days. It is notable that magnesium-doped EGaIn (Mg-EGaIn) exhibits excellent biocompatibility in photothermal therapy. The survival rate of human melanoma cells (C8161) after exposure is close to 100%, and no obvious tissue damage was observed in in vivo experiments. These results confirm that EGaIn and its derivatives have the biological safety basis as tumor treatment carriers and neural interface materials. Li et al. [26] discussed in detail the compatibility characteristics of eutectic gallium-indium alloy (EGaIn) in the biomedical field. In vitro experiments have shown that when EGaIn nanoparticles are co-cultured with 4T1 breast cancer cells and McA-RA7777 liver cancer cells, the relative survival rate of the cells remains above 100%, and they still maintain low toxicity under the action of alternating magnetic fields. The culture experiments of human cells (HeLa cells and adipose-derived stem cells) further verified their biological safety, and no abnormalities were observed in cell morphology and survival rate within 3 days. It is notable that magnesium-doped EGaIn (Mg-EGaIn) exhibits excellent biocompatibility in photothermal therapy. The survival rate of human melanoma cells (C8161) after exposure is close to 100%, and no obvious tissue damage was observed in in vivo experiments. These results confirm that EGaIn and its derivatives have the biological safety basis as tumor treatment carriers and neural interface materials. Based on this, in recent years, research on gallium-based liquid metal flexible electrodes has attracted the attention of researchers.

Jayoung Kim et al. developed a flexible and deformable electrode based on eutectic gallium–indium (EGaIn), overcoming the limitations imposed by the oxide layer on the surface of liquid metals for electrochemical reactions. This system enabled simultaneous detection of biomarkers, such as dopamine (DA) and ascorbic acid (AA). By coating EGaIn with reduced graphene oxide (rGO) to form core–shell particles (REGs) and subsequently loading Pt/Au nanoparticles via a displacement reaction (M-REGs), the catalytic activity was significantly enhanced. The electrode exhibited stable impedance under 30% tensile strain, achieving detection limits of 0.105 μM, 0.065 μM, and 0.068 μM for DA, AA, and uric acid (UA), respectively [27]. The oxidation of liquid metals remains a critical challenge for long-term stability. Interface modification strategies using conductive polymer coatings have proven effective in suppressing metal ion leakage and enhancing electrochemical stability. Huanan Zhang et al. addressed the oxidation issue of gallium-based liquid metals by electrochemically depositing the conductive polymer PEDOT:BF_4_ onto the liquid metal surface, resulting in a 99% reduction in Ga ion leakage as confirmed by ICP-MS analysis. The electrode impedance decreased by three orders of magnitude (from 1 MΩ to approximately 3 kΩ at 1 kHz), and the charge storage capacity was enhanced by 4000 times. In non-human primate (rhesus monkey) models, single-neuron recording achieved a signal-to-noise ratio (SNR) of 21.8, and stable recordings over a four-week period were demonstrated in an earthworm model [28].

#### 2.1.3. Polymer Composite Materials

As key component materials in flexible electrodes, polymers not only act as flexible substrates that perform structural functions for encapsulation layers and hydrogel networks, but some special categories also demonstrate unique conductive advantages. Represented by PEDOT:PSS, conductive polymer materials exhibit excellent ion-electron hybrid conduction properties [29]. Combined with other functional materials, these conductive polymers create synergistic effects, significantly improving the charge-transfer efficiency at electrode interfaces. Furthermore, the chemical functionalization of polymer long chains enables the introduction of specific functional groups. This molecular-level design not only enhances material compatibility in composites but also provides selective responsiveness to target substances, ultimately achieving dual enhancement in both sensitivity and accuracy of electrochemical detection. Polymeric materials play a dual role in flexible electrodes, acting not only as flexible substrates for encapsulation layers and hydrogel networks but also as advanced ionic-electronic conductors when engineered as composites. Specialized polymers, such as poly(3,4-ethylenedioxythiophene):poly(styrene sulfonate) (PEDOT:PSS), demonstrate exceptional interfacial properties through strategic hybridization with complementary materials. The functionalization of polymeric chains with tailored chemical groups enhances composite integration and enables analyte-specific electrochemical responses, thereby improving detection capabilities.

Biocompatible and ionic–electronic conductive polymers have become pivotal in flexible neural interfaces due to a lower modulus compared to the metal base material [30]. Golabchi et al. achieved enhanced electrode longevity and anti-inflammatory performance by integrating dexamethasone-loaded carbon nanotubes into PEDOT matrices, demonstrating their potential in chronic neural recording [31]. Further advancing this paradigm, Cui, X. T. et al. developed a 20-channel polyimide-based microelectrode array (MEA) coated with PEDOT/carbon nanotube composites, achieving a low interfacial impedance of 235 kΩ at 25 Hz. This platform enabled concurrent monitoring of neuronal electrophysiology and dopamine dynamics in murine models, revealing circadian clock gene regulation of dopaminergic signaling pathways [32]. To overcome the limited selectivity of single-component systems, Tahani Mazyad Almutairi et al. synthesized polypyrrole (PPY)/PEDOT:PSS composite via ultrasonic processing. When applied to glassy carbon electrodes, this material detected serotonin across a 0.0083–153.63 μM linear range with 7.4248 μA·μM^−1^·cm^−2^ sensitivity and a 45 pM detection limit, while maintaining > 96% recovery in biological fluids and <5% cross-reactivity to dopamine and ascorbic acid [33]. Conventional hydrogels struggle to balance electrical and mechanical performance. Hyejeong Seong et al. addressed this by functionalizing Ti_3_C_2_T_x_ MXene nanosheets with γ-KH570 silane and crosslinking them with PEGDA-Ca^2+^ networks, creating 3D porous architectures. The resulting hydrogel simultaneously quantified dopamine (2.55 μM limit of detection, 2.5–200 μM range), serotonin (0.83 μM, 1–100 μM), and uric acid (25.11 μM, 10–100 μM) in serum with 81.5–117% recovery and negligible interference. Structural analysis revealed doubled oxidation currents versus planar MXene films and stable operation over 40 days, alongside 80% optical transparency and 2600 mm·s^−1^ air permeability for biocompatible integration [34]. Emerging architectures merge flexible substrates with molecular recognition elements, illustrated by Taylor et al.’s DNA aptamer-functionalized electrodes for real-time cocaine detection in vivo [35].

Here, we summarize the mechanical, electrochemical and service life of several different materials, forming horizontal comparisons between them (Table 1). It is worth noting that the examples of the sensors reported above are usually measured in an ideal buffer liquid system. In vivo tests can be affected by various factors, such as biofouling, scar reactions, and protein interference, which can seriously damage the performance of the equipment. There are already various means, such as functionalizing conductive polymers, electrochemical cleaning, porous coatings [36], and other methods that can reduce the impact of pollution, which will not be discussed here.

**Table 1:** Summary table of key performance comparisons of flexible electrode materials.

Materials	Mechanical Properties	Electrochemical Properties				Lifespan	Advantage	Cite
	Material Resilience	Target Object	Sensitivity	Detection Limit (LOD)	Impedance/Resistance			
GFMEs(rGO)	/	DA	1.54 nA/μM	/	/	/	Anti-pollution	[21]
CFMEs	/	DA	0.41 nA/μM	/	/	/	Can be surface treated	[22]
AuNR@ZIF-8	/	DA	/	0.03 μM	/	/	High selectivity	[23]
pBDD/AuNPs	/	DA	1.5 μA·μM^−1^·cm^−2^	68 nM	/	6 months	Anti-pollution	[37]
rGO/EGaIn/AuNPs	30%	DA	/	0.105 μM	/	/	Stretch impedance stability	[27]
PEDOT:BF_4_/EGaIn	~600%	/	/	/	3 kΩ (1 kHz)	4 weeks	Electrochemically stabilized liquid metal	[28]
EGaIn	480%	/	/	/	3.54 mΩ/square	7000 strain cycles	Temperature and pressure resistance	[38]
GaIn/Pt	100%	/	/	/	250 ± 40 kΩ	2000 strain cycles	Printable	[39]
PPY/PEDOT:PSS/GCE	/	ST	7.4248 μA/μM cm^−2^	45 pM	Rct = 5.21 Ω	100 CV cycles	High sensitivity and high selection	[33]
PEGDA/MXene	/	DA	/	2.55 μM	/	40 days	Breathable and translucent	[34]

While material innovations form the foundation of flexible neural interfaces, clinical translation demands synergistic optimization of tissue-compliant mechanics, multimodal sensing topologies, and anatomically adaptive designs.

### 2.2. Structural Design of Flexible Neuroelectrodes

To achieve efficient and precise neural signal acquisition, the structural design of flexible sensors is critical, in addition to the selection of materials with excellent conductivity and biocompatibility. The structural configuration directly influences the implantation depth, signal acquisition stability, and potential for multimodal functional integration of electrodes within tissues [40]. Depending on the specific application scenarios and the anatomical characteristics of the target tissues, sensor architectures must exhibit high adaptability and flexibility. This is particularly important in complex environments, such as brain tissue, where implantability and minimally invasive requirements must be carefully balanced. In this section, we review two key structural design strategies in flexible neural electrodes: thin-film/gel-based electrodes for multi-site integrated detection and miniaturized penetrating probes capable of deep tissue targeting for high-resolution signal acquisition. These advancements aim to provide systematic solutions for precise monitoring of neural activity across multiple biological scales.

#### 2.2.1. Thin Film Electrodes

In electrochemical sensing applications, thin-film electrodes are widely employed as the platform of choice for three-electrode architectures due to their compatibility with array fabrication and their intrinsic flexibility. For large-area, multisite measurements, these planar devices can be combined with additional sensing modalities to create truly multimodal platforms while exploiting their conformability to biological surfaces [9]. However, conventional thin films often suffer from limited permeability and optical transparency, which can provoke inflammatory responses in adjacent tissues. By integrating a micromesh scaffold with transparent conductive materials, it is possible to reconcile high device performance with enhanced biointegration.

Wang and his team addressed these challenges by fabricating a Ni micromesh core onto which Au nanoparticles were electrodeposited and subsequently coated with PEDOT:PSS, forming a ‘core-shell’ PEDOT:PSS@Au–Ni architecture. By optimizing the Au deposition time (250 s) and PEDOT:PSS coating duration (30 s), they achieved a balance between catalytic activity and electrical conductivity, yielding a dopamine-sensitive electrode with a sensitivity of 1650 µA cm^−2^ mM^−1^, a linear detection range from 1 to 1000 µM, and a limit of detection of 0.27 µM. The electrode maintained excellent selectivity in the presence of common interferents, such as ascorbic acid, uric acid, and glucose, responding exclusively to dopamine. Moreover, the open micromesh design conferred 85% optical transmittance at 550 nm, gas permeability of 2600 mm s^−1^, and robust mechanical flexibility, with negligible performance degradation after 100 bending cycles. Significantly, the combination of laser direct writing and standard electrodeposition processes renders this approach amenable to scalable manufacturing, paving the way for real-time dopamine monitoring in integrated optoelectronic platforms [35].

**Figure 2:**
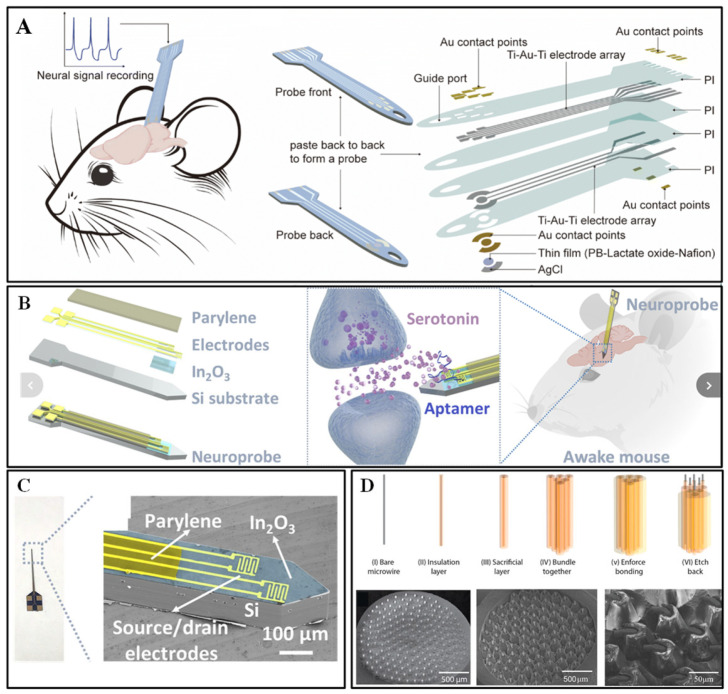
(**A**) Schematic illustration of a flexible multimodal integrated neural probe, including its functional and structural components. Reprinted with permission from [41] Copyright © 2024, RSC Advances. (**B**) Structural and application schematics of a neural probe integrated with aptamer-FET for in vivo neurotransmitter monitoring (e.g., serotonin). (**C**) Scanning electron microscopy (SEM) images of the shaft and tip of a neural probe (150 μm wide × 150 μm thick) featuring two interdigitated FETs. Reprinted with permission from [47] Copyright © 2021, Science Advances. (**D**) Fabrication process of microwire bundles and structural characteristics of their interface with multielectrode arrays (MEA). Upper panel: Microwire bundle fabrication workflow. Lower panel: SEM image of the microwire bundle (wire spacing: 100 μm; wire diameter: ~25 μm). Reprinted with permission from [35] Copyright © 2024, Elsevier.

Thin-film electrodes with a single sensing function seldom satisfy the requirements for multiparametric monitoring. By adopting a double-sided heterogeneous architecture, simultaneous electrophysiological and biochemical interrogation becomes feasible. Shurong Dong et al. developed a flexible neural probe featuring, on one face, a six-channel high-density EEG electrode array (30 × 50 µm electrodes) and, on the opposite side, a lactate electrochemical sensor (overall width ≈ 100 µm; Figure 2A). Chemical gold plating reduced the EEG electrode impedance to an average of 2.57 kΩ at 1 kHz, with stability preserved after three months of saline immersion. The lactate sensor, modified with Prussian blue and catalyzed by lactate oxidase, achieved a sensitivity of 52.8 nA mM^−1^ and displayed negligible response to common interferents, such as glucose and ascorbic acid. A guide port incorporated into the probe head facilitated accurate implantation, and in vivo experiments validated its capability to monitor epileptic electrical signals [41].

The multi-channel simultaneous recording capability of thin-film electrodes endows such devices with the potential to distribute or connect multiple brain regions simultaneously. Wei et al. [42] developed a subcutaneous implanting wireless battery-free device that connects multiple brain region probes through a snake-shaped interconnection structure to achieve multi-channel neural signal recording and regulation. The system employs a flexible printed circuit board (fPCB) integrated with soft platinum black-coated electrodes (PtBk/Au). These thin-film structures (0.9 mm diameter) are mounted on cylindrical polydimethylsiloxane (PDMS) posts for epidural electroencephalography (EEG) recording. Electrochemical deposition of platinum black/polydopamine (PtBk/pDA) composites reduces impedance to 5 kΩ (at 1 Hz) while enhancing mechanical stability. Bilateral optogenetic probes (μ-ILEDs) and drug pumps connect to a central controller through flexible interconnects, supporting independent or synchronized optical stimulation and drug delivery across brain regions. Chronic testing demonstrated stable signal acquisition for 6 weeks, with thin-film electrode performance matching traditional screw electrodes, validating the reliability of flexible thin-film structures for simultaneous multi-region monitoring and modulation. Shang et al. [43] proposed a multi-channel optogenetic platform based on flexible μ-LED arrays, achieving synchronous stimulation of multiple brain regions through distributed optical fibers. The device integrates 5 × 10 micro-LED arrays on polyimide (PI) film substrates (8 μm thick), miniaturized via laser lift-off. Flexible PMMA optical fibers (125 μm diameter) couple to the LEDs using 3D-printed alignment molds, achieving 9% coupling efficiency. Phosphor or quantum dot coatings enable wavelength tuning (460–630 nm), while the ultrathin design allows bending to a 2.25 mm radius of curvature for cortical conformity. Finite element analysis confirms mechanical stability, with serpentine copper interconnects (500 nm thick) sustaining 50% tensile strain while maintaining metal deformation below 0.3%. This design highlights the critical role of thin-film optical components and flexible circuits in enabling programmable multiregional optogenetic stimulation. Yang et al. [44] designed two types of wireless implant devices, head-mounted (HM) and back-mounted (BM), which connected multi-brain region probes through stretchable serpentine interconnects (390 nm thick metal/PI stacks). The HM device integrates bilateral μ-ILED probes for real-time programmable stimulation (in-phase/anti-phase), while the BM version extends to four independently controlled channels. Thin-film metal traces (Ti/Cu/Ti/Au) embedded in PDMS encapsulation (200–800 μm thick) withstand bending radii down to 2.5 mm, accommodating natural movement deformations. Bilateral stimulation experiments demonstrated that synchronized activation of medial prefrontal cortex (mPFC) neurons enhanced social interaction in mice. The integration of thin-film electrodes with flexible encapsulation materials provides mechanical adaptability for independent or synchronized neuromodulation during social behavior studies. Ling et al. [45] adopted an “octopus tentacle” style multi-channel design, integrating μ-leds, microelectrode arrays (with a diameter of 30 μm), and ion-selective sensors through a PI thin-film substrate (25 μm thick). Gold nanoparticle (AuNPs)-modified microelectrodes reduce impedance by 40%, while PEDOT:PSS ion-to-electron transduction layers enhance sensor stability. Four channels implanted in distinct brain regions (e.g., DpG, vHPC) simultaneously record local field potentials (LFPs) and ion concentrations (Ca^2+^/K^+^/Na^+^). The thin-film electrode array supports 16-channel electrophysiological recording (SNR up to 8.1), and ultrathin μ-LEDs (8 μm thick) coupled to optical fibers enable concurrent optogenetic stimulation and calcium dynamics monitoring. Immunohistochemistry confirmed reduced glial activation compared to traditional optical fibers, highlighting the biocompatibility advantages of thin-film/nanostructured interfaces for integrated sensing and stimulation across brain regions. These flexible electronic architectures have achieved breakthroughs in multi-brain region synchronous neural regulation through bionic distributed design. These devices all adopt ultra-thin flexible substrates combined with extensible interconnection structures. Under the premise of maintaining biocompatibility, they support independent or collaborative acquisition and intervention of optical, electrical, and chemical signals in multiple brain regions and achieve dynamic decoding and modulation of complex neural circuits through wireless closed-loop control. The key limitations of traditional rigid devices in spatial resolution, cross-brain region synchronization and long-term implantation adaptability have been successfully solved.

Due to the characteristics of the two-dimensional plane, thin-film electrodes can achieve large-scale and multi-channel acquisition of surface signals. However, limited by the planar layout, there is still a defect of insufficient spatial resolution in the monitoring of deep neural activities. The detection limit is affected by the electrode material and can generally reach the μM level. The type of material and the overall thickness will have a comprehensive impact on the mechanical properties of the film/gel electrode, but generally speaking, they all have good mechanical flexibility. Multiple studies have shown that the factors affecting the biocompatibility of film/gel type electrodes are mainly the intrinsic properties of the material and mechanical flexibility, and have a relatively low correlation with the structure of the film/gel. However, since its implantation method is generally surgical implantation, it will cause a large wound, and large-scale foreign body implantation may lead to an immune response. Therefore, developing thin film/gel electrodes with minimally invasive implantation capabilities is a possible future development direction.

#### 2.2.2. Miniaturized Probes

Miniature probe electrodes are an important structure for electrochemical detection electrodes to acquire signals from deep parts of the nervous system that are difficult to reach with thin-film electrodes. By integrating 50-nm-diameter platinum-iridium nanowires with a flexible polyimide substrate, researchers have developed a miniaturized probe (≤10 μm in diameter) that triples the electrochemically active surface area while maintaining low impedance characteristics (<50 kΩ). Implanted in deep brain regions, this innovative design enables high-sensitivity acquisition of neural signals, providing a breakthrough solution for deep-tissue neuroelectrochemical monitoring [46]. However, the difficulty lies in how to increase the sensitivity while decreasing the probe size and endowing it with a certain degree of multifunctionality. By thinning the material and compositing it with a flexible substrate, it is possible to enhance biocompatibility while maintaining miniaturization.

Anne M. Andrews et al. developed a silicon-based neural probe (150/50 μm thickness) fabricated based on MEMS technology, integrated with In_2_O_3_ nanofilm FETs, and modified with serotonin aptamers on its surface (Figure 2B,C). A femtomolar serotonin detection limit (brain tissue) was achieved, with a selectivity 1000-fold higher than that of interferents, such as dopamine. The dynamic release of serotonin induced by electrical stimulation was successfully detected in a serotonin transporter-free mouse model. The probe achieved enhanced flexibility (27-fold reduction in bending stiffness) by thinning the wafer to 50 μm, maintaining electrochemical performance [47].

The density of conventional microelectrode arrays is limited by processing accuracy. The integration of molecularly imprinted technology with microfilament bundles enables high-density and highly specific dopamine detection. Nicholas A. Melosh et al. integrated molecularly imprinted polymer (MIP)-functionalized microfilament bundles with microelectrode arrays (MEAs) to construct a 2.25 mm^2^ detection area. The microfilament bundle preparation technique enabled the high-density integration of 100 PtIr wires (25 μm diameter) (spacing ≈ 100 μm) (Figure 2D). PEDOT:PSS coating enhances electrochemical activity and combines with dopamine MIP to achieve a detection limit of 820 pM. Previous vivo experiments validated electrical stimulation-induced dopamine release monitoring (brain slice model) [48].

The work of Masvidal-Codina et al. [49] demonstrated that graphene microtransistor arrays significantly enhanced the performance of neural electrodes through their unique disordered nanostructures. This study employed a flexible graphene solution-gated field-effect transistor (gSGFETs) array, whose disordered microelectrode arrangement and two-dimensional material properties directly overcome the voltage drift and high impedance limitations of traditional electrodes when recording ultra-low frequency electroencephalogram (EEG) signals (<0.1 Hz). The electrochemical inertility of graphene (resulting from its low-density state and defect density) and the direct field-effect coupling mechanism have enabled wideband high-fidelity recording from ultra-low frequency (0.1 Hz) to the local field potential frequency band (1 kHz), and high-resolution spatiotemporal propagation maps have been successfully drawn in the cortical diffusative inhibition (CSD) model. At the cellular interface level, the biocompatibility and flexible substrate of graphene support stable cell–electrode coupling, promoting the recording of electrophysiological activities of rat cortical neurons and verifying its potential for monitoring pathological activities in implantable neural electrodes. The work of Lucarini et al. [50] demonstrated that the disordered nanostructure of silicon nanowire (SiNW) pads significantly optimized the function of neural electrodes by enhancing cell–electrode interactions. In this study, a multi-electrode array (NW_MEA) was constructed using disordered, oriented SiNW pads (with diameters of 50–180 nm and lengths of 2–3 μm). The randomly distributed nanowire network guided cell membrane encapsulation and spontaneous intracellular access through physical morphology, achieving stable recording of the action potential of primary cultured neurons in the dorsal root ganglion (DRG). Nanowire mediated mechanical support not only maintained the co-culture growth of DRG neurons and glial cells (verified by GAP-43/GFAP immunofluorescence) but also specifically retained the electrophysiological phenotypes of C fiber injury receptors (such as the “shoulder-shaped” feature of the action potential decline branch), thereby supporting the functional differentiation of cells. Under pharmacological regulation, this platform can continuously monitor the dynamics of the neuron–glial cell network for up to 25 min, highlighting its potential in the research of pain mechanisms and the transformation and application of neural electrodes. In addition to traditional coating and weaving designs, self-assembled interfaces, as an emerging strategy, are capable of forming biocompatible and flexible neural interfaces in vivo. Sha et al. proposed a self-assembled system based on MXene-catalyzed doping, which not only adapts to complex neural structures, but also achieves localized immunomodulation, opening a new possibility for degradable and dynamically adaptive neuroelectronic devices [35]. With the development of novel two-dimensional nanomaterials, composite structure design has become an important strategy to enhance the performance of flexible electrodes. Gou et al. developed a neural electrode based on MXene and PEDOT-PSS composite microfibers, which achieved breakthroughs in electrical conductivity, stability, and multimodal applications. This research fully demonstrated the great potential of nanostructural modulation in the optimization of flexible neural interface materials [51].

Miniaturized probes offer the possibility of high-precision signal capture in deep tissues by reducing device size and increasing integration density, with detection limits reaching the pM level. Although miniaturization and high-density integration have improved spatial resolution, during the implantation process of miniaturized deep brain probes, a hard auxiliary structure is needed to assist in positioning, which inevitably causes damage to the surrounding tissues. This poses a challenge to the compatibility of the probe electrodes in practical applications. In addition, the mechanical stability of long-term implantation still needs further research and further breakthroughs can be made through the collaborative optimization of materials and structures. Future development will focus on the comprehensive improvement of multimodal sensing, adaptive deformation and the stability of biological interfaces.

## 3. Neurotransmitter Sensing Techniques

### 3.1. Electrochemical Detection

Electrochemical (EC) sensors enable quantitative analysis of neurochemicals by detecting current changes induced by chemical reactions, and their core properties are significantly influenced by electrode materials and structures [51]. In recent years, breakthroughs in flexible sensing electrodes have revolutionized EC technology. Ultra-flexible neural electrodes have shown great stability to traditional rigid electrodes in long-term in vivo recordings, mainly due to their better flexibility matching with brain tissue, which significantly reduces mechanical damage. Tao Hu et al. used polyimide (PI) polymer as the substrate material, and the bending strength of the electrode is only about 4.23 × 10^−13^ N-m^2^, which is much lower than that of the traditional silicon-based electrodes, and it is very soft, which can better adapt to the tiny movements of the brain tissues, thus realizing the long-term and stable simultaneous detection of electrophysiological and electrochemical signals [52].

**Figure 3:**
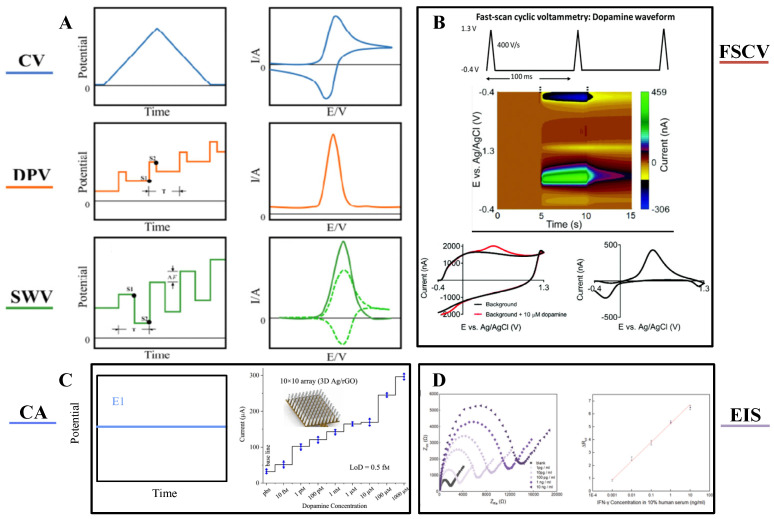
(**A**) Waveforms and responses of common electrochemical analysis techniques: Cyclic voltammetry (CV) voltammogram; Differential pulse voltammetry (DPV) pulse potential waveform; Square-wave voltammetry (SWV) square-wave potential waveform, with distinct sampling positions. The resulting SWV voltammogram (dashed lines) is made up of forward (anodic) and backward (cathodic) components, the net difference of which yields the overall response (solid line). Reprinted with permission from [56] Copyright © 2024, behalf of The Electrochemical Society by IOP Publishing. (**B**) Fast-scan cyclic voltammetry (FSCV) waveform (−0.4 to 1.3 V, scan rate: 400 V s^−1^, frequency: 10 Hz). Pseudocolor FSCV voltammograms for dopamine detection: background current without (black) and with (red) 10 μM dopamine, alongside background-subtracted FSCV voltammogram. Reprinted with permission from [57] Copyright © 2020, Analyst. (**C**) Chronoamperometry (CA) current responses under constant voltage for varying analyte concentrations. Reprinted with permission from [60] Copyright © 2019, Encyclopedia of Analytical Science. (**D**) Electrochemical impedance spectroscopy (EIS) showing linear impedance changes corresponding to different analyte concentrations. Reprinted with permission from [61] Copyright © 2022, Biosensors and Bioelectronics.

A variety of voltammetric techniques have been used to measure neurochemicals, including differential pulse voltammetry (DPV) [53], cyclic voltammetry (CV) [54], and square wave pulse voltammetry (SWV) [55]. As shown in Figure 3A, CV, DPV, and SWV use different waveforms and frequencies to induce oxidation and reduction of compounds. Among these three methods, SWV applies a square wave and the change in current response between forward and reverse pulses captures the difference in each step, thus enabling the detection of different redox actives with higher sensitivity. DPV is relatively sensitive, but its temporal resolution is not sufficient to capture the sub-second changes in some neurotransmitters [56].

**Figure 4:**
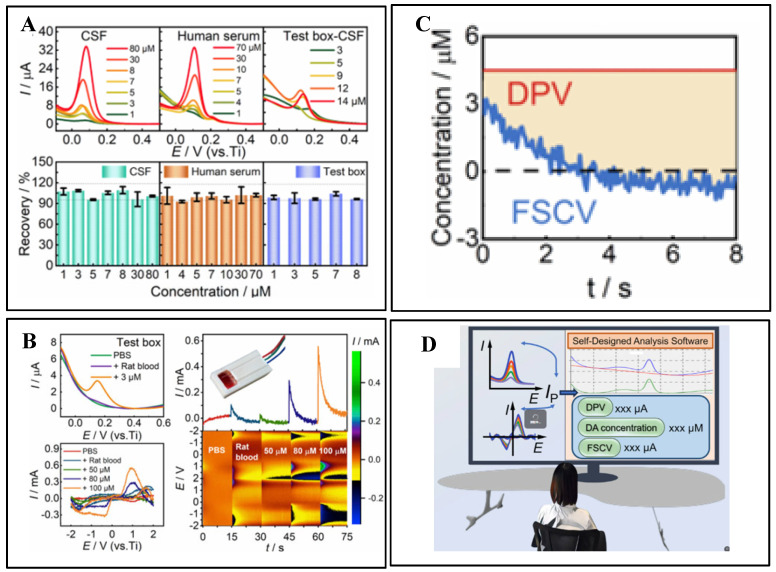
(**A**) Real-time detection of DA. (**B**) DPV, FSCV, and I-T curves measured in a testing chamber, alongside current distribution topology maps. (**C**) DPV and FSCV curves obtained from intracranial implantation. (**D**) Application of a custom-designed real-time analysis software. Reprinted with permission from [53] Copyright © 2022, Elsevier.

Fast Scanning Cyclic Voltammetry (FSCV), which detects neurotransmitters in real time on sub-second time scales. With FSCV, the potential rises from a holding potential to a switching potential and back, Figure 3B typically at a scan rate of 400 V s^−1^ and a frequency of 10 Hz [57]. Gold and platinum co-doped vertical graphene (AuPt-VG) were deposited on the surface of carbon fibers (CFs) to prepare flexible neural microwires for DA detection, and the correlation between differential pulse voltammetry and FSCV was modeled. Figure 4 verifies that the same high sensitivity is exhibited in DPV and FSCV, and FSCV can be used to detect transient DA levels in the brain [53]. Prof. B. Jill Venton’s group at the University of Virginia used the FSCV method to track endogenous dopamine release in specific MB compartments in the adult Drosophila brain during sugar feeding. Acetylcholine stimulation was employed to assess the feasibility of in vivo measurements in awake flies [58]. For selective sensing interface construction, FSCV enables selective detection by applying rapidly changing scanning voltages, with differences in the redox peaks of different neurotransmitters at specific voltages. Zhenan Bao et al. developed NeuroString, a tissue-like stretchable flexible neuroelectrode, by filling graphene/iron oxide nanoparticles into elastomers, which can sense a variety of monoamine neurotransmitters, including DA, norepinephrine (NP), 5-HT, and epinephrine (EP), with stable detection for up to 16 weeks by FSCV [59].

In addition to voltammetry, there are also amperometric methods, of which the most commonly used is chronoamperometry (CA). Figure 3C illustrates the electrochemical oxidation or reduction of an electroactive substance by applying the appropriate potential of a suitable electrode to produce a steady-state anodic or cathodic current [60]. The analysis of target substances by electrochemical impedance spectroscopy (EIS) is also a commonly used detection method Figure 3D, which has the advantages of high sensitivity and low amplitude. A fast biosensor consisting of aptamer/MXene nanosheets on Au microgap electrodes was constructed by Li Ze et al. The high conductivity and impedance sensitivity of MXene were skillfully utilized to realize the rapid construction of the biosensor [61].

Flexible substrates provide a superior microenvironment for biometric components. Bioelectrodes using carbon cloth or polymer substrates can adapt to mechanical deformation and reduce the stress caused by rigid support, thus protecting the active structure of the enzyme with high stability (1 month). Furthermore, flexible composites formed by combining hydrogels (chitosan, polyaniline) with conductive materials (CNT) provide a 3D network structure that maintains a highly hydrated environment and protects the enzyme from dehydration [62]. The chitosan-CNT electrode maintained multi-day stability in in vivo trials, demonstrating the advantages of flexible matrices in dynamic environments. Beyond these direct functional benefits, sustainable design and manufacturing considerations are increasingly important in the development of flexible biointerfaces, like carbon cloth or chitosan-based devices. This includes optimizing fabrication processes to be greener, utilizing renewable and biodegradable materials where possible, and minimizing environmental impact. Szu-Ying Li et al. specifically highlight this approach for neural electrodes, demonstrating a proof of concept for their sustainable manufacturing process, emphasizing its relevance even for sophisticated in vivo applications, such as neural recording arrays [63].

A closed-loop feedback model for PD diagnosis and treatment was recently developed for monitoring changes in DA concentration in the brains of PD model mice and combined with levodopa administration to guide levodopa dosage and injection timing, allowing customization of the drug to improve therapeutic efficacy while avoiding adverse side effects [64]. These advances are driving EC sensors toward miniaturization and intellectualization. Breakthroughs in flexible sensing technology are reshaping the paradigm of neurochemical monitoring, providing entirely new solutions for brain–computer interfaces and neuromodulation [3].

Stability, selectivity, and sensitivity are key metrics for evaluating sensor performance. Table 2 compares these electrochemical detection methods, as well as the differences between flexible and rigid electrodes. Due to the materials, fabrication techniques, and sensing mechanisms used, flexible sensors often exhibit lower performance (e.g., baseline drift) compared to rigid sensors, even without mechanical deformation. Traditional rigid electrodes provide stable signal transduction and integrate well with established circuit designs. However, their mechanical rigidity creates a poor mechanical match with soft neural tissue. This leads to signal instability during physiological movement and makes achieving high spatial resolution over complex brain surfaces difficult. In contrast, flexible electrodes uniquely conform to curved, dynamic tissue structures, achieving minimal mechanical mismatch. This tight structural coupling not only reduces interfacial impedance variations but also increases effective electrode density. Consequently, they enable more localized, high-resolution signal recording. These capabilities are unattainable with rigid systems, especially in highly curved or mobile brain areas, representing an advance in structure and function only possible with flexible platforms. While electrochemical sensors on rigid substrates benefit from mechanical stability and established silicon circuit integration, flexible implementations pose unique challenges. The dynamic deformation of flexible substrates can cause variable electrode-tissue coupling, baseline drift, and strain-induced impedance fluctuations.

**Table 2:** Summary table of DPV/SWV/FSCV flexible and rigid electrodes.

Technology	Electrode Type	Materials	Target Object	Sensitivity	Detection Limit (LOD)	Linear Range	Selectivity	Temporal Resolution	Stability	Robustness	BIOCOMPATIBILITY	Cite
DPV	Flexible	PEDOT–titania–poly(dimethylsiloxane)	EP		100 nM ± 5	20–1000 μM	The EP and DA peaks are placed together	Minute-level		50 consecutive scans, the percentage decrease in current is less than 5%		[65]
	Rigid	Hybrid Multi-walled Carbon Nanotubes-Supernano Diamond	DA	36 ± 2% μA/μM/cm2	9.5 ± 1.2% nM.	33 nM to 1 μM	AA, DA, and 5-HT can be distinguished	Minute-level	5-h electrochemical cycle	Suitable for acute chemical sensing		[66]
SWV	Flexible	PEDOT/CNT	DA		<0.1 µM	0.5–10 µM	Prevents DOPAC, AA, and negatively charged interfering molecules from approaching the electrode surface and generating SWV currents	Second-level		The electrode was placed in the mouse brain and repeated continuously for 90 min	In vivo validation	[67]
	Rigid	Nanoporous diamond/gold particles	DA	0.28 μA/μM	68 nM	3–100 μM	Using Nafion membrane to interfere with AA, L-DOPA, DOPAC, and UA	Second-level	>6 months (room temperature) still retains 95.3% of the average SWV response current	High (20 individual tests (approx. 4% current fluctuation (except for the highest)	Excellent, good stain resistance	[37]
FSCV	Flexible	Metal-complexed polyimide	DA	0.1 nA/fM	5.6 nM	10 nM to 1 μM	High	Millisecond-level			In vivo validation	[59]
	Rigid	CNS–Ta	DA	0.002 nA/µM µm2	8 nM	100 nM–100 μM	Dopamine, uric acid, and ascorbic acid at different potentials	Millisecond-level	10 days	Long-term continuous application of the potential waveform RSD is 3.7% ± 0.8%		[68]
CA	Flexible	rGO/PEDOT:PSS-modified polyimide	DA	15 pA/μM	192 ± 29 nM	1–96 μM	With Nafion membranes, it is possible to resist AA and UA interference, but it is not possible to completely distinguish between NAs	Minute-level	>6 weeks (in vivo)	High (48 weeks electrophysiology)	Excellent (low inflammatory response)	[52]
	Rigid	La/MWCNT	5-HT		13 nM	0.04 µM–0.89 mM	No multiple compounds were found to be likely to interfere	Minute-level	15 days	10 consecutive CV measurements were performed with an RSD < 4.2%		[69]
EIS	Flexible	Au microgap electrodes are made up of aptamers/MXene	IFN-γ		0.26 pg/mL	1 pg/mL to 10 ng/mL	High selectivity for each target protein,	Minute-level	4 days		Well	[61]
	Rigid	Gold electrode/self-assembled monolayer	IgG		0.5 μg/L	5–400 μg/L	High	Minute-level				[70]

### 3.2. Spectroscopic Detection

In addition to the electrochemical methods mentioned above, spectroscopy shows remarkable potential for in vivo brain neurochemistry monitoring, with the advantage of being able to monitor remotely and with high precision. Optical sensing techniques convert signals into measurable parameters by analyzing changes in optical properties caused by object-molecule interactions. The main techniques currently used in this field include fluorescence spectroscopy, near-infrared spectroscopy, Raman spectroscopy, and surface plasmon resonance spectroscopy (SPR). In recent years, breakthroughs in flexible sensing electrodes have provided new integration directions for spectroscopy techniques [71].

**Figure 5:**
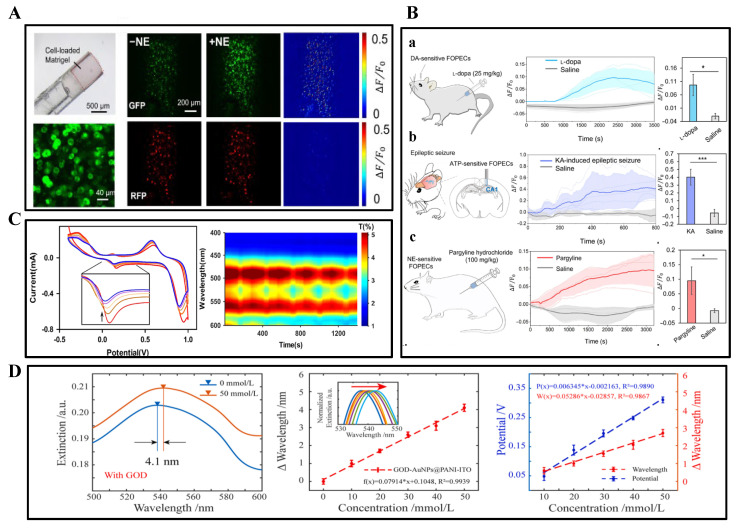
(**A**) Validation of in vitro performance of NE-sensitive FOPECs (plug-and-play fiber-optic sensors). Upon the addition of 100 μM NE, the GRABNE2h sensor (green fluorescence) exhibited significant fluorescence enhancement, while the reference red fluorescence (RFP) remained stable (variation < 0.4%). (**B**) In vivo monitoring of multiple neurotransmitters using fluorescent probes across different animal models/organ systems. (**a**) Experimental diagram of mice under drug (l-dopa) administration. DA dynamics detected with DA-sensitive FOPECs in the hippocampus of mouse brains in vivo after l-dopa or saline is injected intraperitoneally (n = 3). Maximum fluorescence increments due to l-dopa and saline. * *p* < 0.05 (Student’s *t* test). (**b**) Experimental diagram of KA-induced epileptic seizure in mice. Fluorescence dynamics of ATP-sensitive FOPECs in the hippocampal CA1 after intraperitoneal injection of KA or saline (n = 5). Maximum fluorescence increments due to KA and saline. *** *p* < 0.001 (Student’s *t* test). (**c**) Schematic diagram of NE sensing in rat brains in vivo after intraperitoneal injection of pargyline. Fluorescence dynamics of NE-sensitive FOPECs in the LH of rat brains after intraperitoneal injection of pargyline or saline (n = 3). Maximum fluorescence increments due to pargyline and saline administration. * *p* < 0.05 (Student’s *t* test). Reprinted with permission from [72] Copyright © 2023, Science Advances. (**C**) Spectroelectrochemical reaction of DA during CV. Typical CV voltammogram of DA (100 μmol/L) showing current decay with successive CV cycles due to DA depletion in solution. Corresponding spectral responses of nanosensors revealed gradual fading in the red spectral region, consistent with diminished anodic currents. Reprinted with permission from [78] Copyright © 2017, Elsevier. (**D**) Redshift of optical extinction peaks under varying glucose concentrations (0.1–10 mM), demonstrating concentration-dependent spectral modulation (inset is enlarged normalized view of extinction peaks). Reprinted with permission from [77] Copyright © 2022, Elsevier.

When a fluorophore is irradiated to an excited state after absorbing a photon, it emits another photon of a different wavelength when it relaxes back to its original ground state, resulting in a wavelength shift in the absorption–emission spectrum. Fluorescent protein-based sensors have been developed to monitor neurochemical kinetics in vivo. For example, the cell-based neurotransmitter fluorescence-engineered receptor (CNiFER) was developed for volumetric signaling of neurotransmitters, which converts extracellular neurotransmitter signals into intracellular calcium signals and reports [Ca^2+^] using a genetically encoded fluorescent Ca^2+^ sensor. Unfortunately, because CNiFER signals are detected by two-photon fluorescence imaging of implanted cells, sensitive sensing can only be achieved in the superficial layers of the cerebral cortex, limited by the depth of imaging. Neurotransmitter sensors based on G protein-coupled receptor activation (GRAB) and other sensors (e.g., dLight) are used to detect neurochemical dynamics directly in vivo. Following viral infection, fluorescent GRAB neurotransmitter sensors are expressed on cell membranes to indicate neurotransmitters with fast kinetics, ultra-high spatial and temporal resolution, and hyper-specificity. However, the time cost of viral infection usually takes about 2 weeks. Kong et al. prepared a plug-and-play fiber-optic sensor by embedding HEK293 cells expressing the GRAB fluorescent probe into a hydrogel and implanting it into the fiber-optic end face to achieve real-time monitoring of DA, NE, and ATP in non-transgenic animals (mice, rats, and rabbits), as shown in Figure 5A,B [72].

Raman spectroscopy (SERS) leverages molecular fingerprinting properties by analyzing inelastic scattered light to reveal molecular vibrational information. Novel flexible plasmon resonance substrates (e.g., gold nanowires embedded in elastomers) significantly enhance the Raman signal enhancement factor. Combined with serpentine microfluidic channel designs, this enables highly sensitive detection in vivo brain regions, potentially up to the single-molecule level. However, conventional SERS approaches often require the in vivo deployment of plasmonic nanoparticles (like gold nanoparticles) to achieve sufficient signal enhancement at the target site. This raises significant concerns related to nanoparticle accumulation in organs, like the kidneys, and the difficulty in precisely controlling nanoparticle size and distribution over time within complex biological environments, like the brain. Furthermore, the extremely low inherent Raman scattering efficiency results in weak signal intensity and requires extended integration time, limiting the technique’s real-time monitoring capability. To demonstrate neurochemical sensing, Hui Wei et al. assembled glucose oxidase (GOx) and lactate oxidase (LOx) into gold nanoparticles for monitoring glucose and lactate changes in the living brain associated with ischemic stroke via SERS [73]. Separately, Hu et al. developed vibrating fiber photometry as a low-invasive method for in vivo label-free monitoring of biomolecular content in arbitrary deep regions of the mouse brain by spontaneous Raman spectroscopy, bypassing the need for nanoparticles [74]. A promising strategy to mitigate nanoparticle-related safety and control concerns involves using immobilized, biocompatible nanostructures integrated into probes or substrates. For instance, employing safe materials, like gold-coated disordered nanostructures, creates stable hotspots for trapping analytes and sensitive detection within biological tissues, offering a potentially safer in vivo sensing approach [75,76].

Near-infrared spectroscopy (NIRS) enables non-invasive detection by tissue transmission spectroscopy in the 700–1000 nm band, and is particularly suitable for cerebral oxygen saturation monitoring. SPR technology detects molecular binding processes through refractive index changes and can be combined with electrochemical methods to construct multimodal sensing systems. Based on the electrochemical reaction potential modulation of LSPR and combined with the potential scanning LSPR sensing technique, it is applied to the optical sensing detection of single-wavelength electroactive small molecules, as shown in Figure 5D. The catalytic reaction of glucose oxidase on glucose is taken as an example to study the modulation of the LSPR by the enzyme reaction electron transfer [77].

Sensing arrays were designed by combining CV and SPR methods, with good integrity and linearity, promising accuracy for qualitative and quantitative detection even in mixed solutions and brain tissue homogenates. When detecting DA and 5-HT in brain tissue (Figure 5C), the CV-coupled LSPR signal exhibited a better signal-to-noise ratio compared to the CV signal alone, and higher stability compared to LSPR sensing alone [78].

Overall, spectroscopy techniques are promising in the field of brain neurochemistry because of their non-invasive/minimally invasive nature and simultaneous multi-parameter monitoring capability. The flexible sensing technology not only improves the tissue suitability of the optical system but also enhances the signal acquisition efficiency through 3D microstructure design. There are significant differences in sensitivity, spatial and temporal resolution, and operational complexity among the methods, and future development requires breakthroughs in key technological bottlenecks, such as probe development, signal enhancement, and anti-interference, in order to promote their practical applications in in vivo brain monitoring.

In optical sensing, flexible substrates enhance light-tissue coupling, whereas spectroscopic detection methods employing rigid optical probes frequently encounter limitations in optical waveguide integration, alignment maintenance, and avoidance of tissue shadowing. These constraints adversely affect signal strength and restrict penetration depth, particularly in complex or dynamically moving tissues. Flexible optical sensors—fabricated on transparent deformable substrates, like PDMS or SEBS—conformally adhere to curved or mobile surfaces, thereby maintaining optimal optical coupling with minimized light scattering through dynamically adapting curvature.

### 3.3. Sampling Techniques

**Figure 6:**
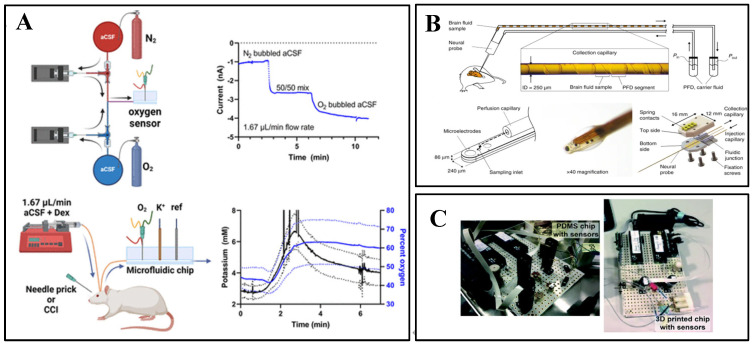
(**A**) Dex-enhanced coMD system for O_2_ monitoring, including O_2_ calibration setup. Probe insertion for monitoring O_2_ and K^+^ in the rat cerebral cortex. K^+^ (black, left axis) and O_2_ (blue, right axis) responses to probe insertion (solid lines are the mean, dotted lines show ±SEM). Reprinted with permission from [82] Copyright © 2023, ACS Chemical Neuroscience. (**B**) Cerebral fluid sampling using neural probes with collection into capillaries. The perfusion capillary is positioned at the distal end of the neural probe, where droplets wet the probe surface, including the sampling inlet. The interface unit incorporates the neural probe clamped between the base and top plates. Reprinted with permission from [77] Copyright © 2017, Nature Communications. (**C**) Simplified modular microfluidic module for continuous and droplet-based calibration systems. Reprinted with permission from [52] Copyright © 2019, Lab on a Chip.

Microdialysis is a sampling technique used to monitor extracellular fluid (ECF) [79]. Intracranial neuromonitoring by microdialysis is approved for clinical use in several countries/regions, and the basis of such techniques has been widely used in neuroscience [80]. In this technique, a catheter inserted into biological tissue is perfused with a solution to allow instantaneous exchange of molecules with ECF through a semipermeable membrane, and the perfusate is then collected and analyzed. This method is widely accepted by the neuroscientist community because of its relative simplicity to make and because it preserves the anatomical and functional integrity of the surrounding tissues [81]. Michael A C, et al. used dexamethasone (Dex) to enhance the ability of microdialysis to monitor interstitial O_2_ and glucose in the cortex of the rat in real-time (Figure 6A), which is critical for patients with traumatic brain injury [82].

In contrast to microdialysis, push–pull probes are systems in which the perfusion solution directly contacts the brain tissue at the tip of the catheter before being recovered. Miniaturized low-flow (10–50 nL min^−1^) push–pull sampling probes have been shown to sustain small amounts of tissue damage in implanted tissue. In addition, a major advantage of this method is that it provides higher collection efficiency compared to microdialysis methods.

Philippe Renaud proposed an in vivo droplet-based sample collection method (Figure 6B) and implanted it in the rat brain to collect fluid samples organized as a series of droplets and to analyze the temporal evolution of elements, such as Na, Mg, K, and Ca, as well as their concomitant neurochemistry, of the neurochemicals captured at a precise location in the brain [83].

In recent years, system miniaturization and microfluidic integration have become valuable. A 3D-printed microfluidic analyzer (Figure 6C) has been developed to monitor dynamic concentration changes in a stream at high temporal resolution. The analyzer can monitor instantaneous metabolite concentration changes as short as 8 s. This high temporal resolution is well suited for monitoring dynamic changes in glucose and lactate in the injured brain [84].

Rigid sampling probes typically require invasive implantation and remain spatially fixed, limiting their ability to adapt to dynamic tissue environments or conduct distributed regional sampling. In contrast, flexible sampling platforms—such as microfluidic membranes or fiber-based probes integrated onto stretchable substrates—can be engineered to conform to anatomical geometries while minimizing tissue trauma. These soft interfaces enable distributed and even dynamically configurable sampling arrangements, facilitating spatiotemporally resolved molecular analysis with reduced immunogenicity. Such bioadaptive sampling strategies are uniquely suited for interacting with living systems where tissue mechanics and architecture dynamically evolve.

## 4. Integration of Multimodal Sensing Systems for Neural Interfaces

With the development of neural interface technology, single-function neuroelectrodes can no longer meet the requirements of in-depth studies of complex neuromodulation mechanisms. Multimodal sensing systems are gradually becoming the future development direction of neural interfaces. The paradigm shift from unidimensional electrical signal detection to multimodal information fusion signifies that the technology is moving towards approaching the inherent complexity of biological systems. Notably, the simultaneous acquisition of electrophysiological and neurochemical signals provides an important breakthrough for elucidating the molecular mechanisms of neuromodulation.

### 4.1. Electrophysiological-Chemical Signal Co-Recording

Traditional electrophysiological experiments typically employ an electrical stimulation combined with an electrical signal acquisition paradigm to validate stimulation efficacy. For electrophysiological–electrochemical co-monitoring systems, synchronized detection of neurotransmitter release dynamics and other specific biochemical substances before and after electrical stimulation can be achieved.

**Figure 7:**
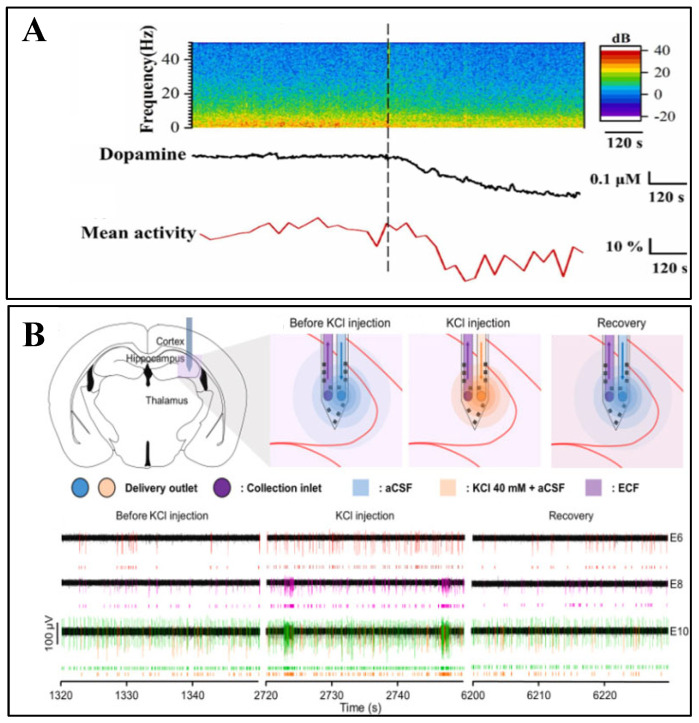
(**A**) (**Top**): Time-frequency spectrogram of LFP power from a representative channel (0–50 Hz frequency range). (**Middle**): Dynamic variations in striatal DA concentration. (**Bottom**): Mean behavioral activity changes in mice before and after 2-methylthio-fenoterol (2MT) administration. Dashed lines indicate 2MT administration timing. Reprinted with permission from [87] Copyright © 2023, Sensors and Actuators B: Chemical. (**B**) Transient electrical signals recorded pre-, during, and post-high KCl infusion and recovery phases following neural activity restoration via drug dilution (6th sampling period). Reprinted with permission from [85] Copyright © 2023, ACS Chemical Neuroscience.

Il-Joo Cho et al. developed a monolithically integrated probe device combining 12-channel electrodes (inter-electrode spacing < 100 μm) with dual microfluidic channels (push–pull sampling). The system features miniaturized dimensions (probe thickness of 40 μm, co-localized sampling area with 200 μm radius). PDMS microfluidic interfaces enable multiplexed drug delivery (glutamate/GABA) and air-isolated sample handling (Figure 7B), permitting simultaneous recording of hippocampal epileptiform discharges (1–15 Hz spectral broadening) and neurotransmitter concentration variations (validated by UPLC-MS/MS) [85].

The successful development of miniaturized probes has established a technical foundation for multimodal detection in deep brain regions, yet challenges persist regarding mechanical compatibility during long-term implantation [30]. To address this critical issue, flexible electronics technology has begun demonstrating unique advantages. Chae U. et al. developed a PI-based ultra-flexible electrode modified with rGO and PEDOT:PSS nanocomposites, which enhanced electrical conductivity, sensitivity, and operational stability. This system enables synchronous detection of electrophysiological signals (e.g., local field potentials and spike activity) and DA concentrations in deep brain regions with high spatiotemporal resolution. The device maintained stable functionality for 6 weeks post-implantation in murine models, exhibiting DA concentration elevation (192 ± 29 nM) coupled with enhanced neuronal firing under pharmacological induction (e.g., nomifensine). This advancement demonstrates significant potential for both fundamental neuroscience research and brain–computer interface development, particularly in neurodegenerative disease studies, such as Parkinson’s disease [52].

Building upon the foundation of long-term stable detection, researchers have shifted focus toward higher spatiotemporal resolution signal analysis to capture transient correlations between neural activity and chemical neurotransmission. Xiaoling Wei et al. developed a parylene-based flexible microelectrode modified with platinum black nanoparticles and PEDOT:PSS, optimized for enhanced electrochemical and electrophysiological performance. This platform enables simultaneous recordings of single-neuron action potentials and DA concentration fluctuations in the striatum (Figure 7A). Following aversive stimulation (2MT-induced) in mice, striatal DA concentrations decreased by approximately 23.5%, accompanied by a 32% reduction in neuronal firing rates and concurrent attenuation of low-frequency local field potential power. This approach reveals the striatum’s role in DA-mediated aversive emotional processing, providing a novel tool for investigating mechanisms underlying psychiatric disorders [86].

With improvements in detection precision, the translation of laboratory achievements into clinical applications has emerged as a new frontier, driving the development of detection systems toward portability and real-time operation. Li, H. et al. developed a boron–nitrogen co-doped graphene-diamond (BNGrD) microelectrode (200 μm diameter) that synergistically combines graphene’s high conductivity with diamond’s stability. This device enables non-invasive scalp electroencephalogram (EEG) monitoring and minimally invasive dopamine detection in biofluids. The system integrates a portable 8-channel EEG headband, electrochemical workstation, and photostimulation glasses, coupled with custom-developed software for real-time analysis of EEG features and dopamine correlations. The device demonstrates an EEG signal-to-noise ratio of 9 dB, a DA detection limit of 124 nM, and exceptional long-term stability (99% performance retention over 48 days). This innovation shows promising potential for remote diagnosis of emotional and cognitive disorders (e.g., depression and schizophrenia) through dynamic correlation analysis between EEG signals and neurotransmitter dynamics [87].

### 4.2. Optical-Electrochemical Co-Monitoring

Electrochemical–electrophysiological co-monitoring has provided critical tools for deciphering neural circuitry, while the emergence of optogenetics has driven innovations in integrating optical modalities with other sensing approaches. To achieve rapid synchronous detection of optogenetic stimulation effects, photostimulation–electrochemical co-monitoring devices enable real-time tracking of specific biochemical substance levels before and after light stimulation.

**Figure 8:**
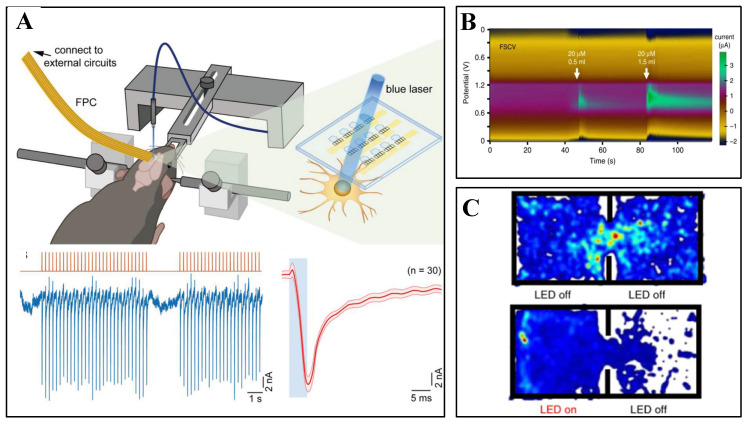
(**A**) In vivo evaluation of ZnO-TFT array by laser stimulating on ChR2 virus expressed on mouse brain. The upper red vertical line segment: the pulse train of optical stimulation applied on the injection cortical surface. Bottom blue line segment: neural signal potential recorded by the transparent ZnO-TFT electrode array exhibiting the light-evoked negative peaks synchronized with the optical stimulation train. Right: averaged light-evoked potential. The blue rectangles illustrate the start and duration of optical stimulation pulses. Reprinted with permission from [88] Copyright © 2023, Advanced Science. (**B**) Fast-scan cyclic voltammetry (FSCV) analysis. Experiments were conducted at room temperature in HCl solution (pH = 4.0) with varying dopamine concentrations, using a saturated Ag/AgCl reference electrode and Pt sheet counter electrode. (**C**) Heat maps of animal positional activity during the pre-test phase (**top**) and under optogenetic stimulation (**bottom**). Warmer hues indicate prolonged dwell durations at corresponding locations. Reprinted with permission from [89] Copyright © 2020, Nature.

Zhang, S. et al. developed a fully transparent (85% optical transmittance) electrocorticography (ECoG) electrode array based on zinc oxide thin-film transistors (ZnO TFTs), overcoming the opacity limitations of conventional active electrodes (Figure 8A top). Figure 8A (bottom) illustrates light stimulation pulse sequences applied to the cortical surface alongside recorded neural potentials, revealing light-evoked negative peaks synchronized with photostimulation patterns. The system exhibits multimodal monitoring capabilities, capturing high signal-to-noise ratio (19.9 dB) slow-wave sleep signals in anesthetized rat brains—superior to gold electrodes (13.2 dB)—while supporting synchronized optogenetic stimulation and electrical recording (SNR 32.2 dB) in transgenic mice. Notably, the device demonstrates artifact-free compatibility with 7T magnetic resonance imaging. This breakthrough represents the first demonstration of highly transparent, active electrodes enabling synchronized multimodal (optical, electrical, MRI) monitoring, providing novel instrumentation for brain science research [88].

The successful development of transparent electrodes has addressed optical pathway obstruction, yet achieving multimodal detection in complex in vivo environments requires further breakthroughs in mechanical adaptability. Zhenan Bao et al. [59] proposed a “NeuroString” flexible biointerface, comprising a metal-composite polyimide laser-induced graphene/nanoparticle network embedded within a styrene-ethylene-butylene-styrene (SEBS) elastomer. This system enables wireless recording of optogenetically induced DA dynamics (sub-second resolution) in the nucleus accumbens (NAc) of mice during fear extinction learning, while simultaneously monitoring neurotransmitter fluctuations. In gastrointestinal applications, it achieves non-invasive 5-HT monitoring during colonic peristalsis, circumventing mechanical irritation caused by conventional rigid probes. Additionally, the platform facilitates inflammation tracking through real-time detection of 5-HT variations in dextran sulfate sodium (DSS)-induced murine colitis models. The device exhibits exceptional mechanical compliance (>1000% stretchability), minimal tissue damage (mild glial response confirmed by immunostaining), and multi-channel multi-analyte detection capabilities. This innovation represents the first wireless synchronization platform for monitoring brain–gut axis neurotransmission, providing novel tools for investigating gut–brain interactions.

Building upon flexible wireless monitoring platforms, the construction of closed-loop regulation systems has become pivotal for enhancing precision in neural interventions, necessitating coordinated integration of multi-physical fields. Xing Sheng et al. developed a multifunctional implantable probe through vertical stacking of miniature blue LEDs (InGaN), diamond films (thermal conduction layer), and PEDOT:PSS electrochemical sensors. A lightweight (2 g) wireless circuit module was engineered to enable remote modulation of photostimulation parameters (frequency, pulse width) and real-time acquisition of dopamine oxidation currents. Dynamic dopamine release in the ventral tegmental area (VTA) was monitored via chronoamperometry (CA) with a current resolution of 0.1 nA. Figure 8B displays fast-scan cyclic voltammetry (FSCV) results, demonstrating current responses to time and voltage following dopamine solution infusion into hydrochloric acid. Figure 8C illustrates optogenetic activation of dopaminergic neurons in the mouse VTA, inducing place preference behavior. The diamond film simultaneously achieves optical transparency (>80%), electrical insulation, and efficient heat dissipation (thermal conductivity > 2000 W/m/K). This work represents the first wireless, multimodal (optogenetic-electrochemical) closed-loop regulation and detection system for deep brain applications [89].

### 4.3. Auxiliary Role of Pressure and Electrophysiological Sensors

Concurrent with the multimodal co-development of optical, electrical, and chemical sensing modalities, synchronous monitoring of physiological pressure parameters introduces a novel dimension for comprehensively understanding neural regulatory mechanisms.

**Figure 9:**
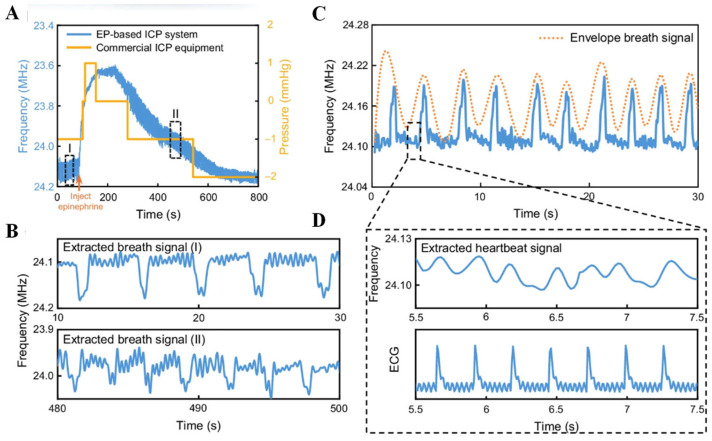
(**A**) Comparative frequency signals captured by the EP-based biotelemetry system and conventional commercial ICP monitors before and after EP injection. (**B**) Respiratory rate variations induced by pharmacological intervention. (**C**) Continuous ICP signal monitoring. (**D**) Extracted heart rate signals validated against standard electrocardiogram (ECG) readings. Reprinted with permission from [90] Copyright © 2024, nature communications.

Luo, J. et al. designed a non-Hermitian circuit based on parity-time (PT) symmetry, achieving enhanced capacitive sensitivity of 115.95 kHz/mmHg (resolution: 0.003 mmHg) through the frequency bifurcation effect in dual-coupled oscillators. The sandpaper-templated fabrication of PVA/H_3_PO_4_ ionomer membranes with surface roughening increased effective contact area, improving pressure-capacitance response (response time: 4.5 ms, cycling stability > 2000 cycles at 10 mmHg pressure). This multimodal device demonstrated intracranial pressure (ICP) monitoring capabilities, validated in rabbit models across physiological pressure ranges (0–10 mmHg). Physiological parameters, including heart and respiratory rates, were extracted from minute ICP fluctuations (0.003 mmHg-level) (Figure 9). The battery-free system operates via external coil coupling for energy transmission and signal backscattering, pioneering the integration of exceptional point physics with iontronics to achieve ultrasensitive, multimodal wireless ICP monitoring [90].

Rigid multimodal neural interfaces often suffer from chronic inflammation, encapsulation, and signal drift due to persistent mechanical mismatch with neural tissue. While they permit high-performance monolithic integration, their biocompatibility remains fundamentally limited. In contrast, flexible systems demonstrate distinct advantages for long-term neural interfacing. Their tissue-matched mechanical modulus significantly reduces micro-motion-induced trauma, maintaining stable electrode-tissue contact over weeks to months. This unique biophysical compatibility enables reliable chronic data acquisition and intervention, which is particularly essential for longitudinal neurochemical or behavioral studies. Unlike rigid devices, flexible platforms preserve the integrity of the neural microenvironment while sustaining high signal quality—an attribute crucial for translating neural engineering applications toward clinical use.

## 5. Closed-Loop Neuromodulation System

Closed-loop neuromodulation systems are progressively redefining the boundaries of neural interface technologies through synergistic “multimodal sensing-intelligent decision-making-dynamic intervention” pathways, transforming real-time detection capabilities of electrical, mechanical, and chemical signals into precise clinical modulation strategies. The breakthrough of such systems lies in their departure from traditional unidirectional signal monitoring limitations, instead leveraging high-dimensional data streams from flexible sensors (e.g., neural electrical activity, chemical microenvironment fluctuations, and tissue mechanical responses) to drive adaptive feedback mechanisms. Recent advances in electrochemical interfaces had real-time detection of neurochemical signals (e.g., dopamine, glutamate) coupled with on-demand therapeutic delivery. For instance, dual-layer conducting polymer films demonstrated subsecond-responsive and spatially confined neurochemical release, fulfilling the core requirements for implementing chemical feedback loops [91]. This enables the implementation of a “sensing-as-therapy” active closed-loop paradigm in scenarios, including epilepsy intervention, motor rehabilitation, and psychiatric disorder management [92].

As the system core, closed-loop diagnostics and therapeutics focus on cross-verification and targeted modulation of multisource biosignals, while machine learning and signal optimization aim to decode dynamic patterns of complex signal coupling. Algorithmic architecture innovations convert raw data into executable modulation commands [16]. The synchronous development of both components not only significantly enhances the spatiotemporal resolution and personalization of diagnosis/therapy but also establishes an intelligent foundation for universal deployment of novel flexible sensing technologies [93].

### 5.1. System Circuitry

Circuit design for flexible sensing systems constitutes a core challenge in realizing high-performance wearable devices and implantable medical instruments. It must simultaneously address signal acquisition precision, anti-interference capability, and energy efficiency while ensuring flexibility and biocompatibility [94]. The circuit architecture typically encompasses sensing units, signal conditioning modules, data transmission units, and power supply systems. Within this framework, the design of the power supply system becomes particularly critical as it directly governs the operational longevity and integration density of the entire system [95].

Signal acquisition in flexible sensors relies on the physical responses of resistive, capacitive, or piezoelectric materials (e.g., strain-induced resistance changes or pressure-modulated capacitor plate spacing). Due to the inherently weak signals and susceptibility to environmental interference—such as temperature drift and motion artifacts—dedicated conditioning circuits are essential for amplification and noise suppression. For instance, Wheatstone bridge configurations leverage differential designs to reject common-mode noise, while instrumentation amplifiers enhance the signal-to-noise ratio in pressure sensing. To address temperature drift, dual-bridge compensation designs substantially reduce thermal errors, as demonstrated in flexible MEMS flow sensors achieving sensitivities of 100 mV/(m·s^−1^) [96]. Filtering circuits, including low-pass RC networks or digital FIR filters, effectively eliminate high-frequency interference, ensuring accurate extraction of physiological signals, such as ECG and EMG [97].

Powering flexible systems necessitates resolving the inherent conflict between energy delivery and mechanical deformation [95]. Flexible lithium-ion or zinc-ion batteries are widely adopted for their high energy density, yet suffer from limited cycling stability and mechanical endurance, as repeated bending frequently causes electrode fracture. Current improvement strategies include developing solid-state electrolytes (e.g., PEO-based polymers) to enhance safety or implementing serpentine wiring layouts to distribute stress [98]. Energy harvesting technologies reduce battery dependency by scavenging ambient energy: piezoelectric materials transduce mechanical vibrations into electricity; triboelectric nanogenerators (TENGs) harvest frictional energy through contact electrification (e.g., at skin-sensor interfaces); thermoelectric materials generate power via body-environment temperature gradients (yielding ~50 μW/cm^2^ at ΔT = 5 K) [97]. These technologies require interfacing with AC-DC conversion circuitry (e.g., full-wave rectifiers) and maximum power point tracking (MPPT) algorithms for efficiency optimization. To address intermittency in single energy sources, integrated harvest–store–distribute modules represent an emerging solution. Hybrid piezoelectric–electromagnetic harvesters, for instance, capture broadband vibrations simultaneously, with rectified outputs stored in flexible supercapacitors, followed by regulated power delivery through buck converters (e.g., LTC3588). Adaptive time-scheduling control strategies can dynamically allocate energy, maintaining only essential monitoring functions during low-activity periods to minimize power consumption [99].

Signal transmission between flexible sensors and external data processing units typically relies on wired connections, but wireless data transfer holds greater practical value. Communication links for such systems can employ RC oscillators, NFC-compliant passive tags, or neural signal modulation techniques utilizing living neurons [94]. While flexible sensors with active circuitry have been monolithically implemented on single substrates, fully integrated monolithic systems encompassing amplification, multiplexing, analog-to-digital conversion, power delivery, and transmission functionalities remain under development [96]. Developing interconnects and encapsulation for flexible circuits requires balancing conductivity and stretchability: Screen-printed silver paste or liquid metals (e.g., Galinstan) offer high conductivity (~10^6^ S/m), yet suffer from cracking under significant deformation. Carbon nanotube/graphene composites maintain conductive pathways through nanoscale network structures, exhibiting minimal resistance change (<5%) even under 30% strain. Encapsulation materials (e.g., polydimethylsiloxane, PDMS) must simultaneously provide electrical insulation, waterproofing, and bioinertness to prevent circuit corrosion from bodily fluids, like sweat [95]. Furthermore, multilayer stacked designs (e.g., sensing layer, circuit layer, power layer) enhance integration density but necessitate shielding layers to mitigate high-frequency interference and prevent inter-layer crosstalk.

Circuit integration for flexible sensing systems requires co-optimization of material properties, and circuit architecture innovation. Future breakthroughs hinge on three critical directions: monolithic integration of solid-state batteries with high-efficiency energy harvesters; development of bio-integrative wireless transmission protocols; and machine learning-based adaptive power management. These advances will accelerate the practical deployment of flexible electronics in wearable healthcare and robotic tactile perception applications [98].

### 5.2. Closed-Loop Diagnosis and Treatment

Closed-loop neural interface systems (CL-NIS) enable real-time adaptive interaction between biological neural circuits and external devices by dynamically resolving biochemical, electrophysiological, and biomechanical signals within neural systems: electrochemical sensing captures neurochemical dynamics (e.g., neurotransmitter concentration fluctuations), electrophysiological sensing decodes electrical signal patterns (e.g., action potentials or local field potentials), while mechanical sensing quantifies physical responses of neural tissue (e.g., pressure or deformation). These three modalities synergistically form an integrated diagnostic framework, enabling closed-loop systems to not only monitor pathological states with high spatiotemporal precision but also trigger context-specific therapeutic interventions.

In vivo dopamine sensing platforms have progressively scaled from single-site probes to high-channel-count arrays. At the simplest extreme, a covalent organic framework–based voltammetric microelectrode achieved nanomolar dopamine detection in the mouse striatum, demonstrating the power of single-sensor sensitivity [100]. Moving to small-scale arrays, flexible microelectrode devices with four to eight carbon-fiber or glassy carbon sites enable simultaneous FSCV at multiple depths or subregions, offering subsecond temporal resolution across distinct loci [66]. Medium-scale platforms—for example, PEDOT/CNT-coated microelectrode arrays integrating up to 16 sensing channels—support concurrent measurement of both tonic and phasic dopamine signals over weeks in freely moving rodents, combining mechanical compliance with chronic stability [101]. Finally, efforts are underway to realize high-density MEAs comprising dozens—or even hundreds—of microelectrodes on soft, conformal substrates, which promise comprehensive spatial mapping of neurochemical dynamics in tandem with electrophysiology for advanced closed-loop neurointerfaces.

Closed-loop neural interface systems dynamically monitor and respond to physiological states by integrating electrochemical, electrophysiological, and biomechanical signals. Neurotransmitter sensing—typically achieved through electrochemical methods—is critical to decoding neurochemical dynamics with high spatiotemporal resolution. However, depending on the temporal window of detection—whether acute (hours to days) or chronic (weeks to months)—the biocompatibility requirements and challenges differ significantly. Below is an in-depth exploration of how these challenges manifest across time scales and how they influence device performance and reliability.

Acute implantation of electrodes can cause various consequences. Within hours of implantation, neutrophils and macrophages infiltrate the implant site, initiating an acute immune response. Pro-inflammatory cytokines and reactive oxygen species are released, which can degrade electrode coatings or interfere with electrochemical sensing accuracy [102]. Typically, this response peaks within the first few days and gradually subsides over 6–8 days [103]. Tissue swelling during the acute phase increases the distance between neurons and electrodes, reducing the quality of both electrophysiological and neurochemical recordings. Swelling can also affect neurotransmitter diffusion dynamics, confounding transient detection fidelity. The biocompatibility issues in the acute detection stage mainly result from mechanical trauma during insertion and initial inflammation.

Over time, the implanted sensor is encapsulated by reactive astrocytes and microglia that form a dense glial scar (typically 50–100 μm thick by week 6) [103]. This barrier isolates the sensor from nearby neurons and significantly increases impedance, reducing signal amplitude and sensitivity [104]. Repeated shearing forces between the stiff probe and soft brain tissue can trigger ongoing inflammatory responses and structural damage. Moreover, long-term protein and lipid adsorption at the sensor–tissue interface can impede neurotransmitter access to the electrode surface. This biofouling effect reduces sensor sensitivity, increases baseline drift, and shortens functional lifespan [105].

To address the biocompatibility challenges inherent to neural sensing, a range of material and structural strategies have been developed to promote stable, tissue-conformal interfaces. Implementing ultrafine, flexible probes—such as carbon fiber electrodes (~7 µm diameter) or polymer-based shanks—dramatically reduces insertion trauma and blood-brain barrier disruption, thereby mitigating immediate inflammatory responses and edema [106]. Biodegradable insertion aids (e.g., PEG, PLGA coatings) temporarily stiffen soft probes for implantation and dissolve to leave a compliant interface [107]. Loading probe surfaces with controlled-release corticosteroids, like dexamethasone, attenuates acute cytokine release and inflammatory cell infiltration. Additionally, biomimetic peptides (e.g., L1CAM, laminin) immobilized on electrodes support neuronal survival and reduce microglial activation within the first two weeks post-implantation [108].

Aiming to solve chronic implantation challenges, such as foreign body response (FBR), gliosis, and mechanical mismatch between the stiff device and soft neural tissue, researchers have developed mechanically compliant substrates—notably polyimide, parylene-C, PDMS, and soft hydrogels—whose elastic moduli more closely approximate brain tissue (~1–30 kPa). Studies show that these compliant materials significantly reduce chronic gliosis and improve neuronal signal stability compared to rigid silicon or metal electrodes [109]. Likewise, hydrophobic coatings on PDMS and polyacrylamide or PDMS coatings over stiffer cores diminish fibrotic encapsulation and immune activation in long-term rodent studies [110].

Composite strategies combining miniaturization, mechanical compliance, and biofunctionalization yield the most promising long-term outcomes. Ultrathin carbon fiber electrodes (<10 µm) embedded in flexible polymer backbones record reliably for over two months with minimal immune response [106]. Moreover, bioresorbable or biohybrid devices—such as transient implants using silk or degradable polymers, or living-electrode constructs integrating biological cells—offer the potential for transient therapeutic windows without long-term foreign body presence [111]. These innovations suggest that future neural prosthetics may harness biomimetic, living, or biodegradable interfaces to achieve seamless, long-term integration with minimal chronic deleterious effects.

#### 5.2.1. Closed-Loop Regulation of Drug Release via Electrochemical Sensing

Electrochemical sensing enables real-time monitoring of neurotransmitter concentration dynamics (e.g., dopamine, glutamate) through microelectrode arrays, providing molecular-level diagnostic evidence for pathological states. The team led by Bin Su developed a covalent organic framework (COF)-modified carbon fiber microelectrode (cCFE) for real-time detection of dopamine (DA) in the brains of Parkinson’s disease (PD) model mice. The nanoporous structure and surface properties (hydrophilicity, negative charge) of the COF coating enhanced anti-biofouling and anti-chemical interference capabilities, improving selectivity and sensitivity. The electrode exhibited a linear current response to DA concentration with a sensitivity of 0.76 nA/μM and a detection limit of 8.2 nM. The cCFE successfully monitored DA concentration dynamics in PD mice brains, guiding L-Dopa dosage and administration timing. Intermittent low-dose drug delivery maintained normal DA levels, prolonging therapeutic efficacy while avoiding side effects (e.g., bradycardia) [64]. Merolla, A. et al. implemented E2GFP as a pH sensor to monitor real-time intracellular acidity changes (chemical signals) and metabolic activity during epileptic seizures, particularly lactate accumulation. Through the chloride ion pump effect of NpHR, neuronal activity was suppressed upon detecting intracellular acidification, effectively reducing seizure duration and severity [112].

#### 5.2.2. Closed-Loop Regulation via Electrophysiological Detection and Stimulation

Electrophysiological sensing employs high-density electrodes to capture electrical signals, such as action potentials and local field potentials (LFPs), revealing spatiotemporal coding patterns of neuronal population [113]. With millisecond-level temporal resolution and multidimensional spatial coverage, this technology serves as a core data source for closed-loop epilepsy intervention and motor intention decoding. The electrode health monitoring methodology proposed in this work is particularly critical for such sensing applications, as compromised electrode–tissue interfaces would distort the recorded neural signals. By implementing residual voltage detection in tandem with electrophysiological recording, we can establish a self-diagnostic neural interface system that ensures both stimulation safety and signal acquisition fidelity.

**Figure 10:**
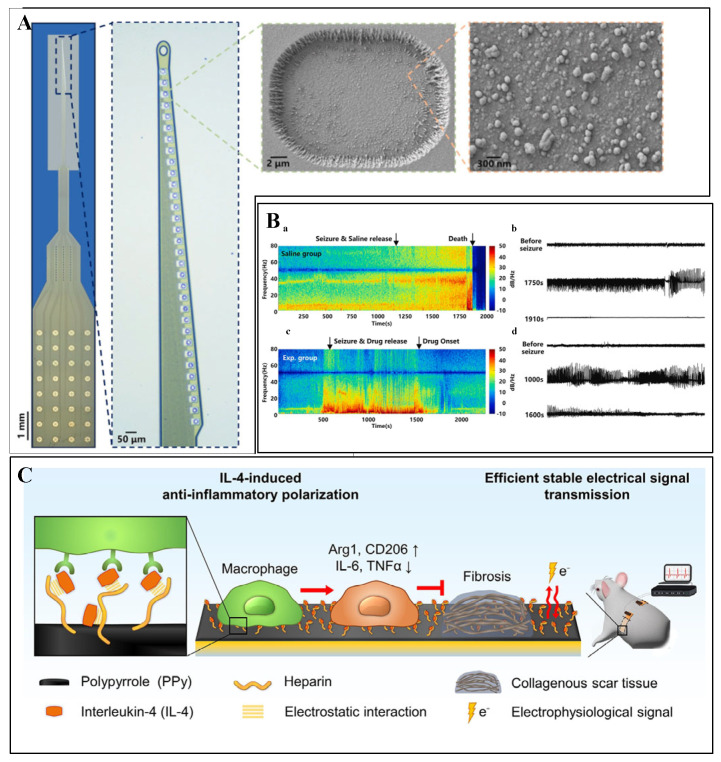
(**A**) Images of flexible electrodes: SEM-acquired views of electrode sites with magnified local site visualization. (**B**) Spectrograms of seizure-related signals from the saline (control) group. (**a**) Spectrogram of the signals during seizure obtained from the saline (no treatment) group. (**b**) Signals from the saline group at different time points. (**c**) Spectrogram of the signals during seizure obtained from the experimental (subcutaneous drug release) group. (**d**) Signals from the experimental group at different time points. Reprinted with permission from [114] Copyright © 2025, Elsevier. (**C**) Schematic diagram demonstrating how IL-4-immobilized PPy/Hep modulates macrophages toward anti-inflammatory phenotypes, reduces scar tissue formation, and enables stable recording of in vivo electrophysiological signals. Reprinted with permission from [118] Copyright © 2023, Acta Biomaterialia.

In recent years, novel strategies for real-time electrophysiological signal detection and closed-loop disease modulation have emerged. Li et al. developed an ultra-flexible Pt-Ir-coated neural probe (Figure 10A), capable of high-sensitivity recording of LFPs and neuronal spike signals, enabling real-time monitoring of epileptic electrographic activity (Figure 10B). Upon seizure detection, the probe delivered 130 Hz biphasic high-frequency electrical stimulation, rapidly terminating 75.16% of seizures, with 90% showing efficacy within 10 s. Concurrently, a subcutaneously implanted electroresponsive drug capsule utilized water electrolysis to generate gas pressure for on-demand diazepam release (5 mg/kg). The drug achieved therapeutic effects within approximately 15 min, providing sustained antiepileptic protection and significantly improving survival rates [114].

Zheng, H. et al. proposed a non-invasive closed-loop acoustic brain–computer interface (aBCI) that detects epileptic seizures in real-time via electroencephalography (EEG), automatically triggers ultrasonic vagus nerve stimulation, significantly reduces seizure frequency and duration, and confirms the critical role of vagus nerve mediation through blockade experiments [115]. Markovi, D. et al. implemented closed-loop neural stimulation with millisecond latency following the detection of pre-seizure iEEG changes using flexible polyurethane depth electrodes integrated with a modular wireless system and adaptive stimulation artifact rejection (ASAR) technology, achieving high-precision, multi-channel real-time intervention [116]. Johnson, D. W. et al. demonstrated the potential of energy-controllable closed-loop intervention by dynamically optimizing low-energy stimulation parameters based on EEG monitoring results to effectively suppress seizure activity [117]. Additionally, Lee, J. Y.’s team designed flexible gold-coated polyimide electrodes for stable subcutaneous ECG signal recording post-implantation. Upon detecting physiological trigger signals, localized IL-4 release was employed to polarize macrophages toward anti-inflammatory (M2) phenotypes (Figure 10 C), reducing TNF-α and IL-6 levels, thereby exploring a novel closed-loop immunomodulation paradigm driven by electrophysiological monitoring [118].

#### 5.2.3. Algorithm-Driven Regulation of Mechanical Signal Sensing

Intracranial pressure (ICP), as one of the most critical mechanical signals in neurocritical care, is closely associated with patient prognosis. Abnormal ICP elevation can restrict cerebral perfusion, induce cerebral hypoxia, and lead to secondary brain injury or mortality [11]. Traditional neuromonitoring techniques predominantly focus on electrophysiological or metabolic parameters, neglecting real-time capture and quantification of mechanical changes, which limits early recognition and intervention in disease progression. To address this gap, Luo, J. et al. developed a flexible iontronic–electronic hybrid sensor using polyimide/metal (Cu/Au) thin-film substrates. By leveraging interfacial capacitance changes in ionic membranes, the sensor achieved high-sensitivity detection of ICP variations (responsivity: 115.95 kHz/mmHg) [90]. This device not only stably records ICP signals but also effectively discriminates respiratory and cardiac interference, significantly improving signal purity and monitoring accuracy. Building on high-fidelity mechanical signal acquisition, Zhang, Z. et al. further proposed a nonlinear autoregressive neural network with exogenous inputs (ANNNARX)-based mean forecasting algorithm (ANNNARX-MFA). Through windowed and subwindowed feature extraction from historical ICP data, this closed-loop rolling prediction model enables precise forecasting of ICP trends across multiple time scales (peak R^2^ = 0.93), outperforming conventional ARMA models and standard ANN approaches [119]. The integration of high-sensitivity mechanical sensing with intelligent predictive algorithms represents a critical advancement toward real-time, adaptive interventions in neurocritical care. This framework also lays the foundation for optimizing signal processing and control strategies through machine learning-enhanced methodologies.

### 5.3. Machine Learning and Signal Optimization

Machine learning transforms raw high-noise, time-varying signals from multimodal sensing data (e.g., electrochemical concentrations, electrophysiological waveforms, mechanical stress) into actionable biomarkers for intelligent interventions. The algorithmic design critically determines whether closed-loop systems can precisely extract biosignal features and drive interventions under complex biological backgrounds.

In multimodal signal analysis, Motamedi-Khozani, R. et al. developed a dual-emissive ratiometric fluorescent probe (BSA-Au NCs/NBS system) combined with linear discriminant analysis (LDA) and partial least squares regression (PLS-R) algorithms. This approach achieved efficient identification and quantification of neurobiomarkers (NEP, EP, DA, HVA, VMA), demonstrating machine learning-assisted multiclass discrimination [120]. For electrochemical signal processing, Zhang, Y. et al. integrated electroactive biofilms (EAB) sensors with Mind Evolutionary Algorithm-optimized artificial neural networks (MEA-ANN), enabling simultaneous detection of multiple contaminants (Cd^2+^, Cr^6+^, TCAA, TCS) in complex aqueous environments. Comparative studies revealed that MEA-ANN outperformed conventional methods with R^2^ > 0.9 [121].

**Figure 11:**
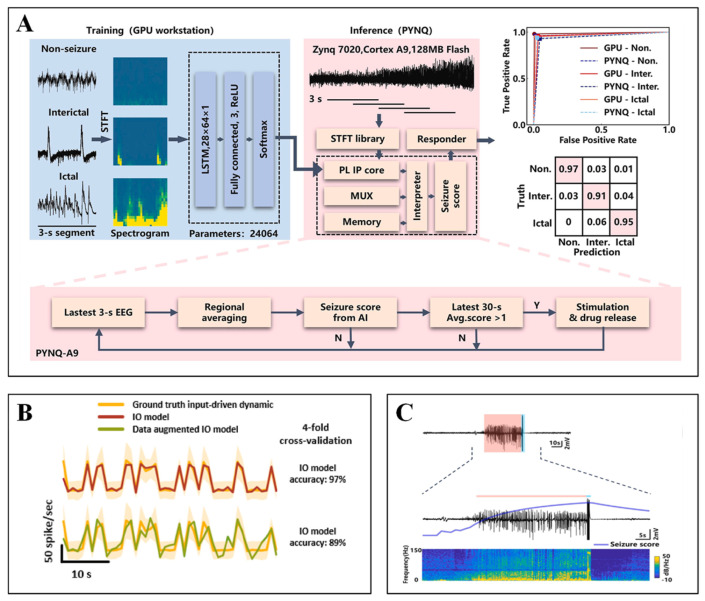
(**A**) Training and recognition processes, accuracy metrics, and modulation logic for autonomous seizure management. Training process of the LSTM network on a GPU and inference process on the embedded system. Post-training ROC curve and confusion matrix from embedded system testing. Logical workflow of BCNIS demonstrating continuous signal acquisition, seizure identification, and execution of neuromodulation. Reprinted with permission from [114] Copyright © 2025, Elsevier. (**B**) Dynamic characteristics of the input–output (IO) model for predicting stimulation-driven neural activity in the NTS. Reprinted with permission from [122] Copyright © 2024, Brain Stimulation. (**C**) Representative recordings of seizure activity under hippocampal control conditions, algorithm-detected seizure events (seizure detection score: blue curve), and corresponding spectrogram. Reprinted with permission from [115] Copyright © 2024, Theranostics.

In real-time neural signal decoding, an edge-TPU-integrated CNN-LSTM system classified memory tasks using multichannel LFP time-series data (F1-score = 69%), highlighting deep learning’s potential in high-dimensional neural data processing [116]. Concurrently, system identification methods were employed to construct linear state-space models (LSSM). Figure 11B illustrates a data-driven approach to characterize stimulation-evoked neural dynamics in the nucleus tractus solitarii (NTS) region, offering novel insights for control strategy modeling [122].

For closed-loop epilepsy neuromodulation, Sun, L. and Zheng, H.’s teams significantly enhanced detection efficiency and response speed through signal optimization algorithms. Sun’s team integrated Butterworth filtering, Welch spectral analysis, and spatial dimensionality reduction, compressing processing latency to 12.3 ms (Figure 11A,C), while employing FP16 quantization to compress LSTM models (58% reduction in computational load, 1.2 mW power consumption) [114]. Zheng, H.’s team developed a multi-threshold hierarchical model that achieved 99% accuracy and millisecond-level response via layered feature extraction. For closed-loop intervention, Sun’s team combined electrical stimulation (75.16% seizure suppression) with sustained drug release, whereas Zheng, H.’s team implemented EEG-triggered ultrasonic neural stimulation via an aBCI system. Both studies collectively reduced epilepsy recurrence rates by 76%, suppressed false positives to 0.3 events/hour, and achieved 0.5 ms-level processing latency [115].

Signal optimization also underpinned the closed-loop design by Markovi, D. et al., who utilized adaptive nonlinear correction (NLC) and ASAR chips to suppress signal artifacts during stimulation. Central 1D histogram equalization (ZHE) further optimized EEG signal distribution, enhancing machine learning input quality [116]. Johnson, G. W.’s team introduced PaCMAP and HDBSCAN for latent space visualization and brain state classification. To address asymmetry in time-series prediction, they innovatively proposed a Circular Minimum Log Hyperbolic Cosine Loss custom loss function, refining the predictive and decision-making performance of closed-loop neuromodulation systems [117].

These studies collectively highlight a trend of multi-level collaborative optimization spanning sensor hardware, signal processing, and algorithmic modeling. They signify the emergence of intelligent closed-loop systems with integrated capabilities in real-time responsiveness, adaptability, and high robustness, laying a solid foundation for future minimally invasive, precise, and efficient neural intervention frameworks.

Rigid closed-loop neuromodulation systems, despite their high computational capabilities, are typically bulky, invasive, and restricted to externalized or tethered configurations. Their form factor and mechanical rigidity confine implantation to planar or skull-mounted surfaces while presenting significant chronic risks, including inflammation, tissue damage, and encapsulation. In contrast, flexible systems enable deep yet minimally invasive implantation within anatomically constrained or highly curved neural structures—such as the spinal cord, brainstem, or enteric plexuses. Their ultrathin, compliant architectures establish intimate tissue contact for bidirectional long-term communication without perturbing the local microenvironment. Critically, flexible platforms monolithically integrate sensing, stimulation, and control elements onto soft substrates, paving the way for fully implantable closed-loop therapeutic platforms—a fundamental capability rigid electronics simply cannot achieve.

## 6. Summary and Perspectives

Flexible sensing electrodes, as a key technology for next-generation neural interface systems, present exciting opportunities for seamless biointegration through soft bioelectronic devices. This review systematically examines recent advancements in material innovation, structural design, multimodal integration, and preclinical applications. Compared to traditional rigid electrodes, flexible counterparts demonstrate superior biological compliance and mechanical conformability, significantly reducing tissue damage and chronic inflammation risks while enhancing signal quality. These attributes provide safer and more efficient solutions for both neuroscience research and BCI systems.

The widespread adoption of novel conductive materials—including graphene, carbon nanotubes, liquid metals, MXenes, and PEDOT:PSS—has endowed flexible electrodes with high conductivity, large specific surface area, and excellent chemical stability. Furthermore, 2D nanomaterials leverage their unique electrical, mechanical, and biocompatible properties to address performance bottlenecks in neural interfaces, driving advancements toward higher sensitivity, lower invasiveness, and enhanced intelligence [123]. Composite material strategies further optimize sensing performance, particularly in electrochemical signal acquisition and multimodal detection scenarios. Concurrently, structural engineering innovations—such as serpentine interconnects, microneedle arrays, nanocrack patterns, and hierarchical interfaces—significantly improve electrode durability and dynamic adaptability within physiological environments.

Flexible sensing electrodes are progressively overcoming single-modality limitations, enabling synchronous acquisition of electrophysiological signals, neurotransmitter dynamics, optogenetic responses, and mechanical stress. By integrating microfluidic chips, optogenetic platforms, and intelligent algorithms, these electrodes now exhibit closed-loop feedback capabilities, demonstrating promising clinical potential in Parkinson’s disease management, seizure prediction, and chronic pain modulation.

Despite these advances, challenges persist. For clinical translation, critical issues include long-term biocompatibility, material stability, controlled biodegradation, and interfacial adhesion. Balancing safety requirements with performance optimization remains inherently contradictory. Additionally, developing high-throughput, low-cost manufacturing methods—ideally leveraging established semiconductor fabrication tools—is essential for scalable production. Current limitations in standardized mass manufacturing processes hinder commercialization. Furthermore, achieving high-density interconnects, wireless powering, low-power signal processing, and remote communication within miniaturized devices imposes stringent demands on system integration.

In minimally invasive applications, exemplified by short-term wearable systems for continuous glucose monitoring, flexible electrodes show broad applicability. These systems typically rely on subcutaneous implants or epidermal contact for physiological signal tracking, necessitating high signal fidelity alongside mechanical flexibility and wearing comfort. Existing solutions still face challenges, such as skin irritation, signal drift, limited lifespan, and motion artifacts during prolonged use or physical activity.

Future development will prioritize intelligentization, systematization, and personalization. Integration with AI algorithms, neural decoding models, and closed-loop control systems may enable “sensing-processing-response” unification. The incorporation of piezoelectric, thermoelectric, and self-powering technologies could reduce external energy dependence, supporting long-term operation in implantable/wearable devices. Personalized structural designs and stimulus-responsive materials will further advance precision medicine and tailored therapeutic interventions.

While flexible sensors offer significant potential for clinical neural interfaces, their translation faces distinct challenges illustrated by ongoing human trials. Systems, like Neuralink (N1 implant), utilize high-density flexible electrode arrays (>1000 channels) for motor decoding, demonstrating feasibility but also highlighting complexities related to long-term biocompatibility, mechanical stability of ultra-fine threads under chronic micromotion, and the need for miniaturized wireless telemetry [124]. Synchron’s Stentrode™, leveraging vascular access to avoid open-brain surgery, exemplifies strategies for reducing invasiveness; however, its fixed intravascular position limits spatial resolution compared to direct cortical interfaces and underscores the trade-off between safety and signal fidelity [125]. The long-standing BrainGate consortium, utilizing rigid microelectrode arrays (Utah array), has achieved multi-year stability for communication in paralysis, but reports signal drift and glial encapsulation, reinforcing the need for flexible interfaces with enhanced chronic biocompatibility [126]. These clinical efforts collectively emphasize critical translational hurdles for flexible sensors: ensuring chronic mechanical/electrochemical stability, mitigating foreign body responses that degrade signals, achieving robust wireless power/data transfer in compact biocompatible formats, and establishing standardized manufacturing for clinical-grade devices.

In conclusion, flexible sensing electrodes are poised at a critical juncture of technological maturation and clinical translation. Their multidimensional sensing and intelligent interaction capabilities promise to redefine neuroscience research paradigms and catalyze human–machine integration ecosystems. As core components of neuroelectronic systems, their strategic importance will continue to grow, warranting sustained interdisciplinary exploration and collaborative innovation.

## Data Availability

Not applicable.
